# T cell‐inflamed gene expression profile is associated with favorable disease‐specific survival in non‐hypermutated microsatellite‐stable colorectal cancer patients

**DOI:** 10.1002/cam4.5429

**Published:** 2022-11-07

**Authors:** Hang Yin, Tabitha A. Harrison, Sushma S. Thomas, Cassie L. Sather, Amanda L. Koehne, Rachel C. Malen, Adriana M. Reedy, Michelle A. Wurscher, Li Hsu, Amanda I. Phipps, Syed H. E. Zaidi, Polly A. Newcomb, Ulrike Peters, Jeroen R. Huyghe

**Affiliations:** ^1^ Institute for Public Health Genetics University of Washington Seattle Washington USA; ^2^ Public Health Sciences Division Fred Hutchinson Cancer Center Seattle Washington USA; ^3^ Genomics Resource, Fred Hutchinson Cancer Center Seattle Washington USA; ^4^ Experimental Histopathology, Shared Resource, Fred Hutchinson Cancer Center Seattle Washington USA; ^5^ Vaccine and Infectious Disease Division Fred Hutchinson Cancer Center Seattle Washington USA; ^6^ Department of Biostatistics University of Washington Seattle Washington USA; ^7^ Department of Epidemiology University of Washington Seattle Washington USA; ^8^ Ontario Institute for Cancer Research Toronto Ontario Canada

**Keywords:** colorectal cancer, gene expression profile, somatic mutation, survival, T‐cell inflammation

## Abstract

**Background:**

The anti‐tumor immune response plays a key role in colorectal cancer (CRC) progression and survival. The T cell‐inflamed gene expression profile (GEP) is a biomarker predicting response to checkpoint inhibitor immunotherapy across immunogenic cancer types, but the prognostic value in CRC is unknown. We evaluated associations with disease‐specific survival, somatic mutations, and examined its differentially expressed genes and pathways among 84 sporadic CRC patients from the Seattle Colon Cancer Family Registry.

**Methods:**

Gene expression profiling was performed using Nanostring's nCounter PanCancer IO 360 panel. Somatic mutations were identified by a targeted DNA sequencing panel.

**Results:**

The T cell‐inflamed GEP was positively associated with tumor mutation burden and microsatellite instability high (MSI‐H). Higher T cell‐inflamed GEP had favorable CRC‐specific survival (hazard ratio [HR] per standard deviation unit = 0.50, *p* = 0.004) regardless of hypermutation or MSI status. Analysis of recurrently mutated genes having at least 10 mutation carriers, suggested that the T cell‐inflamed GEP is positively associated with *RYR1*, and negatively associated with *APC*. However, these associations were attenuated after adjusting for hypermutation or MSI status. We also found that expression of genes *RPL23*, *EPCAM*, *AREG* and *ITGA6*, and the Wnt signaling pathway was negatively associated with the T cell‐inflamed GEP, which might indicate immune‐inhibitory mechanisms.

**Conclusions:**

Our results show that the T cell‐inflamed GEP is a prognostic biomarker in non‐hypermutated microsatellite‐stable CRC. This also suggests that patient stratification for immunotherapy within this CRC subgroup should be explored further. Moreover, reported immune‐inhibitory gene expression signals may suggest targets for therapeutic combination with immunotherapy.

## Graphical abstract

T cell‐inflamed gene expression profile score, a biomarker predicting immune checkpoint inhibitor responses, is related to colorectal cancer (CRC)‐specific survival among non‐hypermutated and microsatellite stable patients, a group refractory to immunotherapy. Our finding suggests that patient stratification for immunotherapy within this CRC subgroup should be explored further.

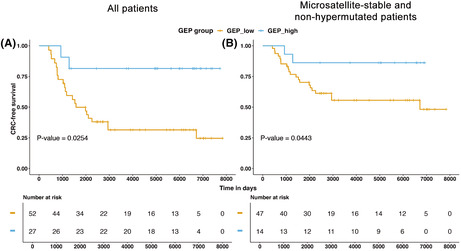

## 1 INTRODUCTION

Colorectal cancer (CRC) is the third leading cause of cancer‐related death in the United States.^1^ The patient's anti‐tumor immune response plays an important role in tumor progression and survival. Immunotherapeutic strategies, such as checkpoint blockade inhibitors, harness this immune response and are revolutionizing cancer treatment. However, only a subset of patients respond to immunotherapy, and underlying factors are incompletely understood. A high tumor mutation burden^2^ and high microsatellite instability (MSI‐H)^3^, ^4^ are favorably associated with immunotherapy treatment response. These indirect measures of tumor neoepitope burden have also been associated with favorable survival regardless of treatment.^5^ The presence of T cell infiltration in the tumors is another important biomarker predictive of clinical outcome.^6^ However, T cells exhibit diverse functional states in the tumor microenvironment and anti‐tumor immunity is a dynamic multiple‐step process involving the release of chemokines, interferon signaling, and expansion of CD8^+^ cytotoxic T cells.^7^ Therefore, an integrative biomarker comprehensively capturing the immune response is needed to characterize patients and predict prognosis.

Recently, a signature called the T cell‐inflamed gene expression profile (GEP) has been developed that predicts response to PD‐1 blockade therapy using pembrolizumab.^8^ Based on RNA from baseline tumor samples, the T cell‐inflamed GEP is composed of 18 interferon‐gamma‐responsive genes related to antigen presentation, chemokine expression, cytolytic activity, and adaptive immune resistance. Jointly with tumor mutation burden, it has shown good predictive performance for progression‐free survival and overall survival (OS) after anti‐PD‐1 treatment in pan‐cancer clinical datasets.^9^ In an analysis of The Cancer Genome Atlas (TCGA) pan‐cancer dataset, the T cell‐inflamed GEP was not statistically significantly prognostic for CRC but was modestly prognostic for melanoma.^10^

Our study aimed to evaluate the prognostic value of the T cell‐inflamed GEP in CRC patients. We computed the T cell‐inflamed GEP score using newly generated Nanostring nCounter gene expression profiling data from formalin‐fixed paraffin‐embedded (FFPE) tumor blocks of 80 CRC patients in the Seattle Colon Cancer Family Registry. The prognostic value of the T cell‐inflamed GEP was evaluated by examining its association with CRC‐specific survival using Cox proportional hazards models adjusting or stratifying for known prognostic factors like MSI and hypermutation status. We then evaluated its association with somatically mutated genes and pathways. Lastly, we identified genes and pathways that were differentially expressed with the T cell‐inflamed GEP.

## 2 METHODS

### 2.1 Study participants

This study included 84 incident CRC cases from the Seattle Colon Cancer Family Registry^11^ with long‐term clinical follow‐up, including survival data, and FFPE primary tumor tissue blocks collected between 1997 and 2003. CRC diagnosis, anatomical site, and stage, vital status, and cause of death were confirmed by pathological records, medical records, cancer/death registries, and/or death certificate information. Patients with a hereditary CRC syndrome diagnosis were excluded from this study. All participants provided written informed consent where appropriate and this study was approved by the relevant research institutional review board.

### 2.2 Gene expression profiling

We performed expression profiling of 770 genes using Nanostring's nCounter PanCancer IO 360 panel (NanoString Technologies). From each FFPE block, we cut one fresh 4 μm section for hematoxylin and eosin (H&E) staining and three fresh 7 μm sections for RNA extraction. After histopathologist review and marking of tumor lesions on H&E slides, we macrodissected tumor tissue from the three sections. We extracted total RNA using the Qiagen RNeasy FFPE kit (Qiagen). Firstly, all paraffin was removed by treatment with Deparaffinization Solution (Qiagen). On‐column DNase I digestion and RNA extraction were carried out following the manufacturer's recommended protocol. We quantified RNA using RiboGreen (Invitrogen) and assessed RNA integrity by Agilent 4200 TapeStation analysis (Agilent). TapeStation results were used to calculate DV200 values, the percentage of RNA fragments >200 nucleotides, which were used to determine RNA input amount for the Nanostring nCounter assay. Gene expression was measured using the Nanostring nCounter platform and protocol following manufacturer's recommendations (NanoString Technologies).

### 2.3 Data quality control and normalization

We performed quality control (QC) for binding density, image quality, positive control linearity and limit of detection to all raw data using the nSolver 4.0 Analysis Software (NanoString Technologies). Raw expression data were normalized to External RNA Controls Consortium control probes to eliminate technical variability, then were normalized to a list of stable housekeeping genes by subtracting the average of the log_10_ counts of the housekeeping genes from the log_10_ count of each of the genes. Housekeeping genes used for normalization were selected by the geNorm algorithm.^12^ Samples with low counts or outlying mean squared error values for reference genes, which indicate poor normalization quality, were excluded from further analyses. All 84 attempted samples passed all QC metrics. After subsequent normalization of expression data, we removed four outlying cases because of low‐quality normalization (*n* = 2) or low counts (*n* = 2). In total, this study included 80 CRC cases that were included in the analysis.

### 2.4 T cell‐inflamed GEP score and immune cell type score

We calculated the T cell‐inflamed GEP score using the weighted sum of normalized expression values of 18 genes related to antigen presentation, chemokine expression, cytotoxic activity, and adaptive immune resistance: *PSMB10*, *HLA‐DQA1*, *HLA‐DRB1*, *CMKLR1*, *HLA‐E*, *NKG7*, *CD8A*, *CCL5*, *CXCL9*, *CD27*, *CXCR6*, *IDO1*, *STAT1*, *CD274* (*PD‐L1*), *CD276* (*B7‐H3*), *LAG3*, *PDCD1LG2* (*PDL2*) and *TIGIT*, as previously described.^8^, ^9^ The continuous score was then standardized to have a mean of 0 and a standard deviation (SD) of 1. The categorical score was dichotomized into T cell‐inflamed GEP^Hi^ (upper tertile) and T cell‐inflamed GEP^Low^ (middle and lower tertile). From the previous work,^8^, ^9^ most people with a score below the top tertile had rapid disease progression. The abundance of immune cell populations was quantified using the average of the log_2_‐transformed expression values for sets of marker genes that are expressed stably and specifically in given cell types^13^ and implemented in the nCounter Advanced Analysis 2.0 module of Nanostring's nSolver software.

### 2.5 Tumor DNA sequencing and somatic mutation calling

We analyzed existing somatic mutation data. Sample and sequencing details, as well as somatic mutation calling, were described previously.^14^ Briefly, we conducted targeted deep sequencing on DNA from tumor tissue macrodissected from the same FFPE block and matching normal tissues using a custom AmpliSeq sequencing panel of 205 genes.

### 2.6 Statistical analyses

#### 2.6.1 Survival analyses

We performed multivariate Cox proportional hazards regression using the R survival package^15^ to test the association of the continuous and dichotomous T cell‐inflamed GEP score with CRC‐specific survival. Person time accrued from the date of diagnosis to the date of death or the end of follow‐up. Patients who died from causes other than CRC were censored at the date of death. We examined proportional hazards assumptions by testing for a nonzero slope of the scaled Schoenfeld residuals as a function of survival time. Primary analyses of survival were not adjusted for stage at diagnosis to account for the fact that meaningful associations could operate via an impact on disease aggressiveness and, therefore, an impact on stage. We also performed an analysis adjusted for a grouped stage variable constructed by combining stage I and II patients (*n* = 35), and stage III and IV patients (*n* = 44). Since patients in this study received standard‐of‐care treatment based on stage at diagnosis, we used stage as a proxy for treatment.^16^ Additional covariates and stratified analyses are described below. As a secondary analysis, we analyzed overall survival as the outcome.

#### 2.6.2 Somatic mutation analyses

We used linear regression to test the association between the continuous T cell‐inflamed GEP score and somatic mutations. We analyzed 11 cancer driver genes with at least 10 mutation carriers in our data: *APC*, *TP53*, *KRAS*, *SYNE1*, *RYR1*, *FBXW7*, *SMAD4*, *PIK3CA*, *AMER1*, *GNAS*, *KMT2C*. Additionally, we analyzed five mutated signaling pathways with at least 10 mutation carriers in our data: Wnt, transforming growth factor‐beta (TGF‐beta), receptor tyrosine kinases (RTK)/RAS, tumor protein p53, and IGF2/phosphatidylinositide 3‐kinases (PI‐3‐kinase). Multiple comparison correction was done separately for mutated genes and mutated pathways, both using the Benjamini‐Hochberg method.^17^

#### 2.6.3 Differential expression and gene set enrichment analyses

Differential expression analysis was performed in all cases and the subset of non‐MSI‐H and non‐hypermutated cases. The predictor was the continuous T‐cell inflamed GEP score. The analysis was performed using the negative binomial model, as implemented in Nanostring's nSolver Advanced Analysis Module. Pre‐ranked gene set enrichment analyses (GSEA) was performed with the GSEA software version 4.1.0.^18^ Gene sets comprised 25 common signaling pathways related to tumor characteristics, microenvironment, and immune response that were represented on the PanCancer IO 360 panel (Table S1). Multiple hypothesis adjustment was performed using the Benjamini‐Yekutieli method.^19^ Genes and gene sets with false discovery rate (FDR)‐adjusted *p*‐value <0.05 were considered significantly expressed.

#### 2.6.4 Covariate adjustments and stratified analyses

All analyses were adjusted for age at diagnosis, sex, and hypermutation or MSI status. Survival analyses were also conducted among the subset of non‐hypermutated or MSS cases, adjusting for age at diagnosis, sex, and tumor mutation burden.

All statistical analyses were performed using R statistical software version 4.0.3 unless stated otherwise. Multiple testing corrections were performed using the R built‐in function p.adjust().

## 3 RESULTS

### 3.1 Descriptive statistics

This study included a total of 84 CRC cases. After normalization of expression data, we removed 4 outlying cases because of low‐quality normalization (*n* = 2) or low counts (*n* = 2), so 80 cases were taken forward for analysis. The demographic and clinical characteristics of the study participants are summarized in Table 1. The average age at diagnosis was 59.5 years and numbers of males (*n* = 41) and females (*n* = 39) were similar. The majority of patients were non‐hypermutated (82.5%) and MSS (82.5%). Most cases were diagnosed with tumors in the proximal colon (47.5%) and at stage III (44.3%). After a mean follow‐up of 4035 days (11 years), a total of 46 deaths occurred and 24 (52.2%) of these were attributed to CRC. Among patients who died of CRC, about half were diagnosed at the distal colon (45.8%) and at stage III (52.2%).

**TABLE 1:** Characteristics of study participants

Characteristics	All cases		Cases who died of CRC	
	Number	%	Number	%
Total	80	‐	24	‐
Age at diagnosis (years)				
≤54	21	26.25	5	20.83
55–64	27	33.75	9	37.50
≥65	32	40.00	10	41.67
Mean (range)	59.5 (25–74)		61.3 (38–74)	
Sex				
Male	41	51.25	7	29.17
Female	39	48.75	17	70.83
MSI status				
MSI‐H	14	17.50	1	4.17
MSS	66	82.50	23	95.83
Hypermutation status				
Hypermutated	14	17.50	1	4.17
Non‐hypermutated	66	82.50	23	95.83
Cancer site				
Proximal colon	38	47.50	8	33.33
Distal colon	23	28.75	11	45.83
Rectal	15	18.75	4	16.67
Other	4	5.00	1	4.17
Cancer stage				
Stage I	14	17.72	3	13.04
Stage II	21	26.58	2	8.70
Stage III	35	44.30	12	52.17
Stage IV	9	11.39	6	26.09
T‐cell inflamed gene expression profile score				
High	27	33.75	2	8.33
Low	53	66.25	22	91.67

Abbreviations: CRC, colorectal cancer; MSI, microsatellite instability; MSS, microsatellite‐stable; MSI‐H, microsatellite instability high.

**FIGURE 1:**
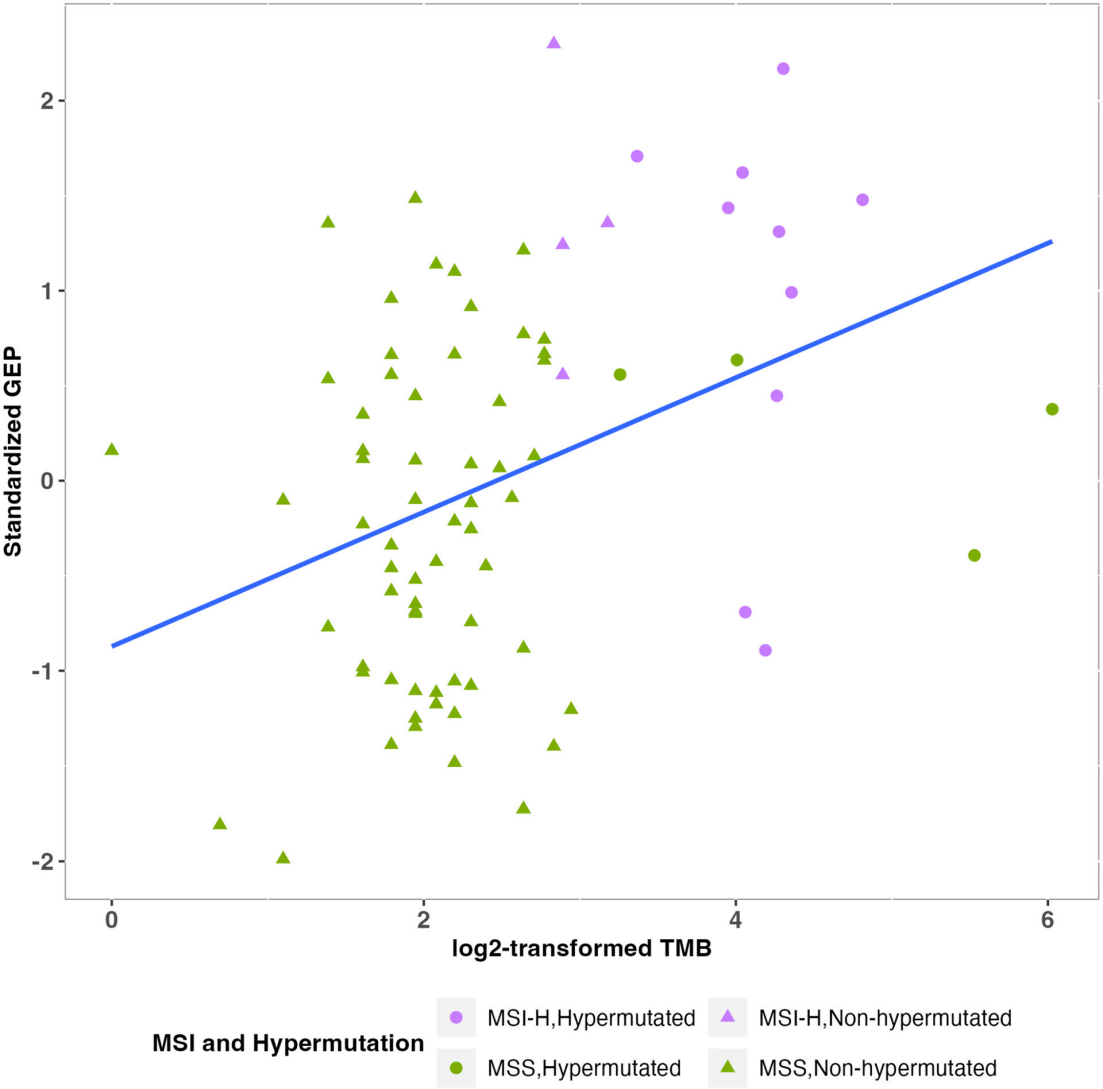
Distribution of standardized T cell inflamed‐gene expression profile across log_2_‐transformed total number of mutations (Pearson *r* = 0.37, *p* = 0.0008).

The T cell‐inflamed GEP score ranged from −0.892 to 0.459 with mean of −0.265 (SD = 0.315) and median of −0.269, and approximately followed a normal distribution (Figure S1). The T cell‐inflamed GEP score was positively correlated with total mutation burden (Pearson *r* = 0.37, *p* = 0.0008), hypermutation status (*p* = 0.001), MSI‐H (*p* = 2.67 × 10^−6^) (Figure 1; Figure S2), and abundance scores for most immune cells except for mast cells (Figure S3).

### 3.2 Survival analyses

**FIGURE 2:**
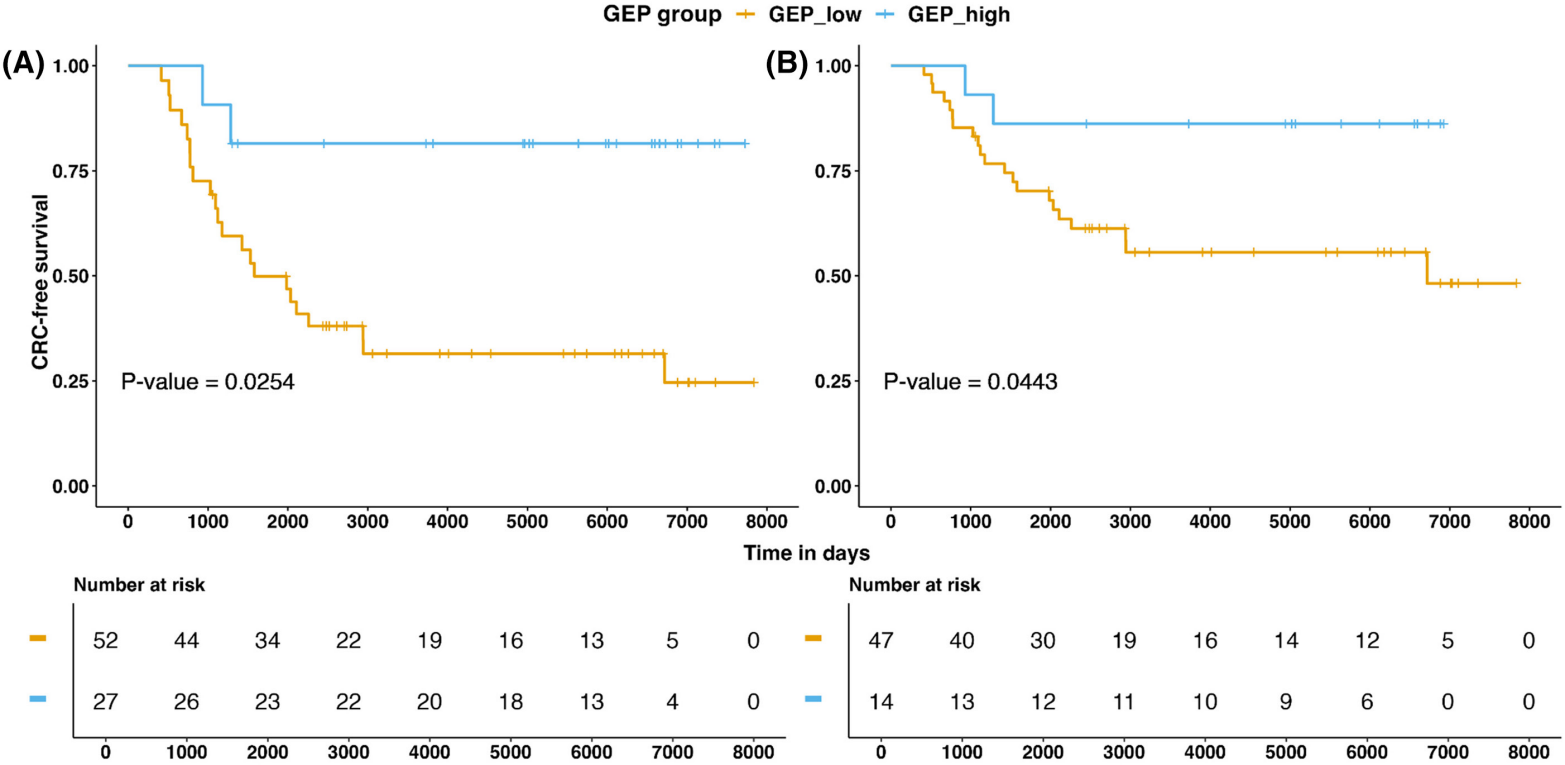
Kaplan–Meier plot of the association between colorectal cancer‐specific survival and dichotomized T cell‐inflamed expression profile among (A) all cases and (B) non‐hypermutated and microsatellite‐stable cases.

Associations between continuous standardized T cell‐inflamed GEP score and CRC‐specific survival are summarized in Table 2. Cases with higher T cell‐inflamed GEP score had more favorable CRC‐specific survival (hazard ratio [HR], per 1‐SD unit change of the score = 0.50, 95% confidence interval [CI] = 0.31–0.80, *p*‐value = 0.004) after adjustment for age at diagnosis, sex, hypermutation or MSI status, and total mutation burden. The effect of hypermutation or MSI status on CRC‐specific survival was attenuated after adjusting for the T‐cell inflamed GEP score (unadjusted HR = 0.08, 95% CI = 0.01–0.62, *p*‐value = 0.015; adjusted HR = 0.17, 95% CI = 0.02–1.37, *p*‐value = 0.1). Among the subset of non‐hypermutated and MSS cases, the standardized T cell‐inflamed GEP score also showed prognostic value for CRC‐specific survival (HR = 0.53, 95% CI = 0.33–0.85, *p*‐value = 0.008), and this association remained significant after adjusting for log‐transformed total mutation burden (HR = 0.35, 95%CI = 0.19–0.66, *p*‐value = 0.001). Adjusting for stage in these analyses did not appreciably affect coefficient estimates and *p*‐values (Table S2) and hence did not alter conclusions. We found that the prognostic value of T cell‐inflamed GEP score also applied to overall survival (Table S3). We observed similar results when dichotomizing the score into T cell‐inflamed GEP^Hi^ and T cell‐inflamed GEP^Low^ cases. The T cell‐inflamed GEP^Hi^ group had more favorable CRC‐specific survival (HR = 0.19, 95% CI = 0.04–0.81, *p*‐value = 0.025) (Figure 2; Table S4) and overall survival (HR = 0.47, 95% CI = 0.22–0.98, *p*‐value = 0.044).

**TABLE 2:** Associations between continuous standardized T cell‐inflamed GEP score and CRC‐specific survival

Analysis	No. cases	No. events	Covariate adjustments	HR (95% CIs)	*p*‐value
All cases	79^a^	24	Model 1	0.41 (0.26, 0.64)	7.84E‐05
			Model 2	0.50 (0.31, 0.80)	0.004
			Model 3	0.42 (0.25, 0.70)	0.000946
Non‐hypermutated and MSS cases	61	23	Model 1	0.53 (0.33, 0.85)	0.008
			Model 2	‐	‐
			Model 3	0.35 (0.19, 0.66)	0.001

*Note*: Model 1: adjusted for age at diagnosis and sex. Model 2: adjusted for age at diagnosis, sex, hypermutation or MSI status. Model 3: adjusted for age at diagnosis, sex, log‐transformed total number of mutations.

Abbreviations: CI, confidence interval; CRC, colorectal cancer; GEP, gene expression profile; HR, hazard ratio; MSI, microsatellite instability; MSS, microsatellite‐stable.

^a^ One case with missing time to event, so the number of cases dropped to 79.

### 3.3 Somatic mutation analyses

Next, we evaluated if somatic mutations were related to the T cell‐inflamed GEP scores. Among the 11 single genes and five pathways with 10 or more mutation carriers, the standardized T‐cell inflamed GEP score was negatively associated with *APC* mutation status, while positively associated with mutation status of the genes *SYNE1*, *RYR1* and *AMER1*, and the RTK/RAS pathway (Table 3). After FDR adjustment, only the association for *RYR1* remained significant (16 carriers, FDR‐adjusted *p*‐value = 0.028), and the *APC* mutation was suggestively significant (44 carriers, FDR‐adjusted *p*‐value = 0.054). However, after adjusting for hypermutation and/or MSI status these two associations were no longer significant. The results for all analyzed genes and pathways are given in Table S5.

**TABLE 3:** Associations between continuous standardized T cell‐inflamed GEP score and somatic mutations

Gene/pathway	No. carriers	Covariates for adjustment 1		Covariates for adjustment 2	
		Nominal *p*‐value	Adj *p*‐value^a^	Nominal *p*‐value	Adj *p*‐value^a^
*APC*	44	0.0098	0.0538	0.0934	0.4648
*SYNE1*	16	0.0396	0.1190	0.1993	0.5438
*RYR1*	16	0.0025	0.0276	0.1123	0.4648
*AMER1*	10	0.0449	0.1190	0.4415	0.6349
RTK_RAS^b^	35	0.0153	0.0764	0.0889	0.4447

*Note*: Covariates 1 includes age at diagnosis and sex. Covariates 2 includes age at diagnosis, sex, hypermutation or MSI status.

Abbreviation: adj, adjusted; MSI, microsatellite instability.

^a^ Nominal *p*‐value is adjusted by false discovery rate methods.

^b^ RTK_RAS pathway included any mutations in genes *BRAF*, *ERBB2*, *ERBB3*, *KRAS*, *NRAS*.

### 3.4 Differential expression and GSEA

**FIGURE 3:**
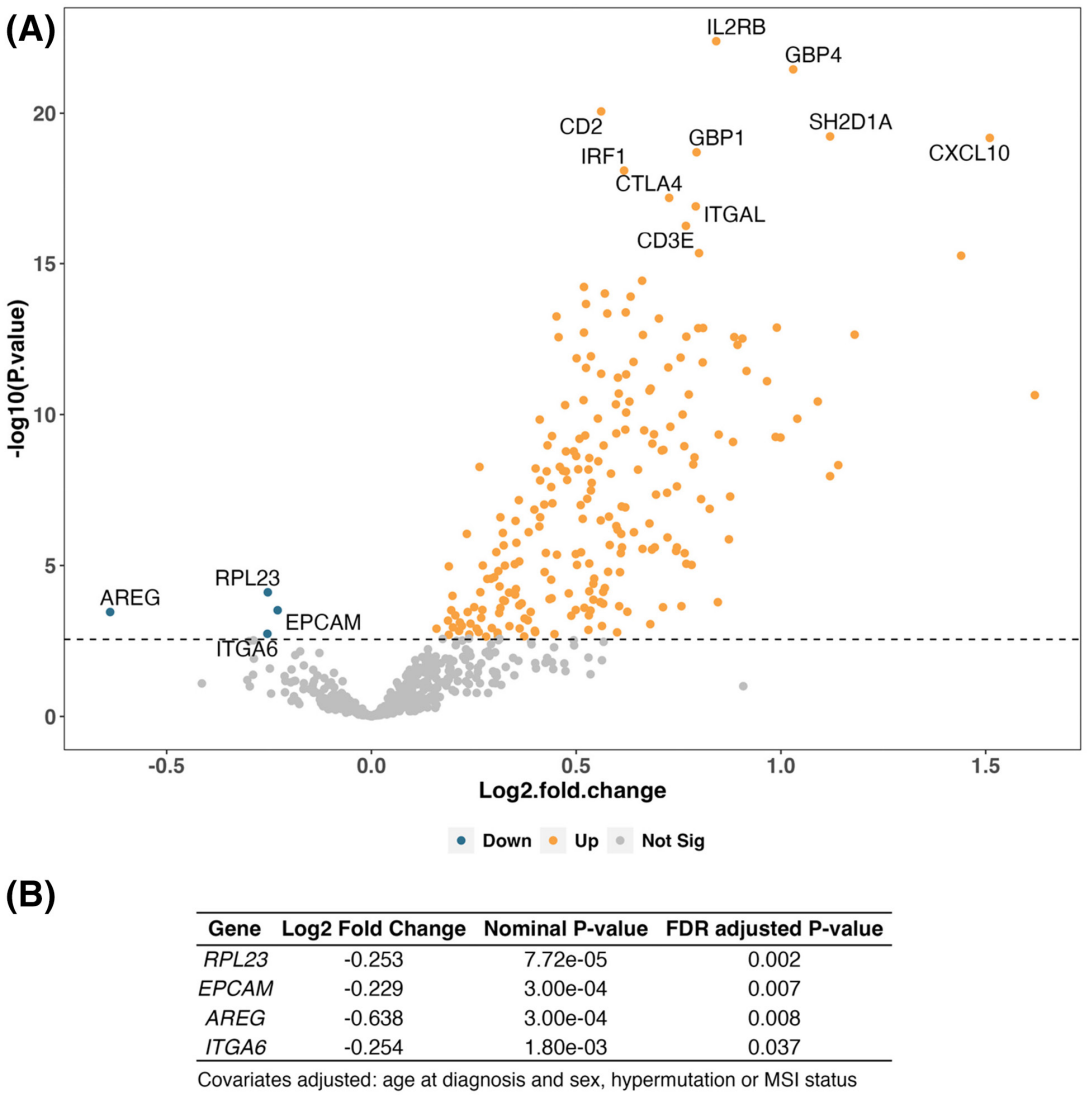
Volcano plot (A) and summary table for negative associations (B) of genes differentially expressed across continuous T cell inflamed‐gene expression profile.

Finally, we explored whether expression of genes and pathways were associated with continuous T cell‐inflamed GEP scores. Excluding genes used to calculate the T‐cell inflamed GEP score, we observed many more positively than negatively associated genes, with stronger effect sizes for positively associated genes (Figure 3). Among genes with negative associations, the genes *RPL23*, *EPCAM*, *AREG*, and *ITGA6* had significant associations (FDR adjusted *p*‐value <0.05) after adjusting for hypermutation and MSI status (Figure 3B). In the GSEA analysis, Wnt signaling was down‐regulated for continuous T cell‐inflamed GEP scores (FDR adjusted *p*‐value = 0.015), and the lymphoid compartment and cytotoxicity gene sets were up‐regulated. However, associations for Wnt signaling and cytotoxicity were no longer significant after adjusting for hypermutation and MSI status (Figure S4; Table S6).

## 4 DISCUSSION

### 4.1 Prognostic value of the T cell‐inflamed GEP score

This study investigated the prognostic value of the T cell‐inflamed GEP score, a predictive biomarker for clinical response to anti‐PD‐1 immunotherapy, in CRC patients who received other treatments (surgery and chemotherapy). We found that a higher T‐cell inflamed GEP score was associated with MSI‐H status, higher tumor mutation burden, and favorable CRC‐specific survival. The T‐cell inflamed phenotype predicted cancer‐specific survival independent of MSI‐H and hypermutation status, and the association remained statistically significant among the subset of MSS and non‐hypermutated patients. Adjustment for tumor stage, a good proxy for treatment during the time period covered by this study,^16^ did not qualitatively change the results of these analyses. The prognostic value of the T cell‐inflamed GEP is perhaps not surprising as this transcriptional signature integrates multiple facets of the complex intratumoral T cell adaptive immune response.^8^, ^9^, ^20^, ^21^ However, to our knowledge, the prognostic relationship with CRC‐specific survival has not been reported before.

In their pan‐cancer analysis of the TCGA, Danaher et al.^10^ reported a null association between the T cell‐inflamed GEP score, which they called tumor inflammation signature in their paper, and OS in colon adenocarcinoma (COAD) samples. A possible explanation for these inconsistent findings may be that the TCGA COAD sample comprises more early stage tumors than our sample.

### 4.2 T cell‐inflamed GEP and somatic mutations

We found that the standardized GEP score was positively associated with somatic mutations in *RYR1* and negatively associated with somatic mutations *APC*. The negative association for *APC* has been reported previously by Cristescu et al.^9^ However, we found that these associations were no longer significant after adjusting for hypermutation or MSI status. This could be explained by the fact that driver mutations in *APC* are enriched in MSS and non‐hypermutated tumors^14^ and, hence, hypermutation and/or MSI status is therefore a confounding variable. Yet, biallelic *APC* mutations have been previously associated with increased Wnt signaling and decreased T cell infiltration in both MSS and MSI‐H CRC tumors by us^22^ and in other cancer types.^23^, ^24^ *RYR1* encodes ryanodine receptor 1.^25^ Experiments in mice models suggest carriers of some gain‐of‐function mutations in *RYR1* have faster immune responses in malignant hyperthermia.^26^ The somatic mutation rate of *RYR1* in CRC is higher than other cancer types,^27^ but the enhanced immune function has not been reported.

### 4.3 T cell‐inflamed GEP and differentially expressed genes and gene sets

In the differential expression analyses and GSEA, most significantly associated genes and pathways showed positive correlations with the T cell‐inflamed GEP score that were stronger than for genes and pathways with negative associations. This is expected because expression of immune cell genes tends to be positively correlated with an adaptive immune response.^10^ Here we focus on negatively expressed genes and pathways because these could represent immune inhibitory mechanisms that may suggest potential therapeutic targets. Among the four significantly negatively expressed genes, *EPCAM* encodes epithelial cell adhesion molecule, which is a frequently expressed tumor‐associated antigen^28^ and has been detected in colorectal adenocarcinomas.^29^, ^30^, ^31^, ^32^ Amphiregulin is one of its ligands and is encoded by *AREG*, which was also significantly negatively expressed (discussed further below). The binding of amphiregulin to the extracellular domain of EPCAM (EpEX) activates epidermal growth factor receptor signaling, which promotes tumor cell survival^33^ and immune evasion.^34^ Additionally, the overexpression of *AREG* and *EPCAM* is associated with tumor progression and unfavorable survival outcomes in multiple cancer types.^29^, ^34^, ^35^ Anti‐EpCAM treatment like EpAb2‐6 could induce apoptosis in colorectal tumor cells,^35^ and its combination with atezolizumab, an anti‐PD‐L1 antibody, enhances the cytotoxic activity of CD8^+^ T cells.^34^ This biological evidence supports our findings that *EPCAM* and *AREG* are important genes inhibiting adaptive immune responses. However, their therapeutic potential for CRC has not been fully proven by two clinical trials investigating *EPCAM* targets Adecatumumab^36^ and Edrecolomab.^37^, ^38^ Further investigation of the role of *EPCAM* may provide new insights into drug development or therapeutic combination with immunotherapy.^39^

The other two genes negatively associated with the T cell‐inflamed GEP score were *ITGA6* and *RPL23*. *ITGA6* encodes integrin alpha 6 (α6). Integrins are heterodimeric transmembrane proteins primarily expressed by epithelial cells^40^ and regulating cell proliferation and differentiation.^41^ Expression of genes in the integrin gene family is correlated with immune infiltrates including macrophages, memory CD8^+^ T cells, and exhausted T cells.^42^ Gene *ITGA6* enhances eosinophils specifically.^30^, ^42^ Overexpression of its subunit, *ITGA6A*, was observed in proliferative colorectal adenocarcinoma cell lines.^43^ Finally, *RPL23* encodes ribosomal protein L23, a component of the 60 S ribosomal subunit that is widely expressed across tissues.^30^ Overexpressed *RPL23* has been reported to suppress tumor growth in lung cancer,^44^ but it is an unfavorable prognostic biomarker in ovarian cancer.^45^ Its role in CRC remains unclear.

The transcription of the aforementioned, negatively associated *AREG* gene is regulated by the Wnt signaling pathway,^46^ which was negatively enriched in the GSEA analysis for continuous T cell‐inflamed GEP. Wnt signaling is a tumor‐intrinsic oncogenic pathway that suppresses antitumor immune response.^23^, ^24^ In a large multi‐omics analysis of CRC, we previously reported that activated Wnt signaling is associated with biallelic *APC* mutations and the absence of T‐cell infiltration in both MSI‐H and MSS CRC tumors.^22^ We reported that the T cell‐inflamed GEP was negatively associated with *APC* mutations and Wnt signaling pathway but both associations were no longer significant after adjusting for MSI status. One explanation is the differences in expression of Wnt signaling component genes between MSI‐H and MSS tumors,^47^ and biallelic *APC* mutations are the principal driver in MSS tumors. Wnt signaling is a potential therapeutic target. Several Wnt antagonists or modulators in combination with chemotherapy are under preclinical or clinical investigation for CRC treatment.^48^

### 4.4 Study limitations

Our study has several strengths, including the availability of long‐term clinical follow‐up data with complete disease‐specific mortality information, in combination with primary tumor GEP and detailed somatic mutation data on a comprehensive and validated panel of CRC driver genes, and on MSI‐H and hypermutation status. In the investigation of suppressive mechanisms, we fully adjusted for the elevated neoantigen and enhanced immunogenicity driven by MSI‐H and accumulation of somatic mutations, and identified genes associated with the T cell inflamed GEP. Despite these strengths, our study was limited by the small sample size. In the somatic mutation analyses, we only analyzed genes with a sufficiently high mutation count. Also, it has been reported that CRC is a heterogeneous cancer with different immune landscapes depending on the anatomical subsite where the tumor arises.^49^ Therefore, subsite‐stratified analyses of the T cell inflamed GEP based on larger sample sizes are warranted.

## 5 CONCLUSIONS

Our study showed that the T cell‐inflamed GEP is a prognostic biomarker for sporadic CRC, even in MSS and non‐hypermutated patients. The T‐cell inflamed GEP complements traditional tumor‐intrinsic predictors like hypermutation and MSI status, and together they give a more comprehensive picture of anti‐tumor immune activity in CRC. We discovered somatic mutations and genes and gene sets with expression values associated with this score. The immune‐inhibitory signals reported in our study may suggest new leads for tractable drug targets. Results also suggest that patient stratification for immune checkpoint inhibitor treatment within MSS and non‐hypermutated CRC patients, which comprise the majority of CRC patients, should be investigated further.

## AUTHOR CONTRIBUTIONS

**Hang Yin:** Conceptualization (equal); data curation (equal); formal analysis (lead); methodology (equal); software (lead); visualization (lead); writing – original draft (equal); writing – review and editing (equal). **Tabitha A. Harrison:** Conceptualization (equal); formal analysis (supporting); methodology (supporting); project administration (supporting); writing – original draft (supporting); writing – review and editing (supporting). **Sushma S. Thomas:** Investigation (supporting); resources (supporting); writing – review and editing (supporting). **Cassie L. Sather:** Data curation (supporting); investigation (supporting); resources (supporting); writing – review and editing (supporting). **Amanda L. Koehne:** Investigation (supporting); resources (supporting); writing – review and editing (supporting). **Rachel C. Malen:** Investigation (supporting); project administration (supporting); resources (supporting); writing – review and editing (supporting). **Adriana M. Reedy:** Investigation (supporting); project administration (supporting); resources (supporting); writing – review and editing (supporting). **Michelle A. Wurscher:** Investigation (supporting); resources (supporting); writing – review and editing (supporting). **Li Hsu:** Formal analysis (supporting); funding acquisition (equal); methodology (supporting); writing – review and editing (supporting). **Amanda I. Phipps:** Resources (supporting); writing – review and editing (supporting). **Syed H. E. Zaidi:** Resources (supporting); writing – review and editing (supporting). **Polly A. Newcomb:** Funding acquisition (equal); resources (supporting); writing – review and editing (supporting). **Ulrike Peters:** Conceptualization (equal); formal analysis (supporting); funding acquisition (equal); methodology (supporting); resources (supporting); writing – original draft (supporting); writing – review and editing (supporting). **Jeroen R. Huyghe:** Conceptualization (equal); data curation (equal); formal analysis (supporting); funding acquisition (equal); methodology (equal); project administration (lead); software (supporting); supervision (lead); visualization (supporting); writing – original draft (equal); writing – review and editing (equal).

## FUNDING INFORMATION

This work was supported by the National Cancer Institute at the National Institutes of Health (R21CA230486 to J.R.H., P20CA252733, U01CA137088, R01CA248857, U01CA167551, U01CA74794, R01CA076366, R01CA118699, R01CA107333, K05CA142885 to P.A.N., R01CA189532 to L.H.), and through cooperative agreements with members of the Colon Cancer Family Registry and Principal Investigators. This work was in part funded by an Investigator Initiated Sponsored Research Agreement between Fred Hutch's Immunotherapy Integrated Research Center and Juno Therapeutics (SRA180603). This research was supported by the Genomics and Experimental Histopathology Shared Resources of the Fred Hutch/University of Washington Cancer Consortium (P30 CA015704). Scientific Computing Infrastructure at Fred Hutch was funded by ORIP grant S10OD028685.

## CONFLICTS OF INTEREST

The authors declare no potential conflicts of interest.

## ETHICS APPROVAL STATEMENT

All participants provided written informed consent where appropriate and this study was approved by the relevant research institutional review board.

## Supporting information


Appendix S1

## Data Availability

The DNA sequencing data analyzed during this study are available at the database of Genotypes and Phenotypes (dbGaP, accession phs002050.v1.p1). Gene expression and relevant other data are available upon reasonable request from the corresponding authors. The data are not publicly available due to privacy or ethical restrictions.

## References

[1] Siegel RL , Miller KD , Fuchs HE , Jemal A . Cancer statistics, 2022. CA Cancer J Clin. 2022;72:7‐33. 35020204 10.3322/caac.21708

[2] Yarchoan M , Hopkins A , Jaffee EM . Tumor mutational burden and response rate to PD‐1 inhibition. N Engl J Med. 2017;377:2500‐2501. 29262275 10.1056/NEJMc1713444 PMC6549688

[3] Mlecnik B , Bindea G , Angell HK , et al. Integrative analyses of colorectal cancer show immunoscore is a stronger predictor of patient survival than microsatellite instability. Immunity. 2016;44:698‐711. 26982367 10.1016/j.immuni.2016.02.025

[4] Le DT , Durham JN , Smith KN , et al. Mismatch repair deficiency predicts response of solid tumors to PD‐1 blockade. Science. 2017;357:409‐413. 28596308 10.1126/science.aan6733 PMC5576142

[5] Gryfe R , Kim H , Hsieh ET , et al. Tumor microsatellite instability and clinical outcome in young patients with colorectal cancer. N Engl J Med. 2000;342:69‐77. 10631274 10.1056/NEJM200001133420201

[6] Galon J , Costes A , Sanchez‐Cabo F , et al. Type, density, and location of immune cells within human colorectal tumors predict clinical outcome. Science. 2006;313:1960‐1964. 17008531 10.1126/science.1129139

[7] Chen DS , Mellman I . Oncology meets immunology: the cancer‐immunity cycle. Immunity. 2013;39:1‐10. 23890059 10.1016/j.immuni.2013.07.012

[8] Ayers M , Lunceford J , Nebozhyn M , et al. IFN‐γ‐related mRNA profile predicts clinical response to PD‐1 blockade. J Clin Invest. 2017;127:2930‐2940. 28650338 10.1172/JCI91190 PMC5531419

[9] Cristescu R , Mogg R , Ayers M , et al. Pan‐tumor genomic biomarkers for PD‐1 checkpoint blockade‐based immunotherapy. Science. 2018;362:eaar3593. 30309915 10.1126/science.aar3593 PMC6718162

[10] Danaher P , Warren S , Lu R , et al. Pan‐cancer adaptive immune resistance as defined by the tumor inflammation signature (TIS): results from The Cancer Genome Atlas (TCGA). J Immunother Cancer. 2018;6:63. 29929551 10.1186/s40425-018-0367-1 PMC6013904

[11] Newcomb PA , Baron J , Cotterchio M , et al. Colon Cancer Family Registry: an international resource for studies of the genetic epidemiology of colon cancer. Cancer Epidemiol Biomarkers Prev. 2007;16:2331‐2343. 17982118 10.1158/1055-9965.EPI-07-0648

[12] Vandesompele J , De Preter K , Pattyn F , et al. Accurate normalization of real‐time quantitative RT‐PCR data by geometric averaging of multiple internal control genes. Genome Biol. 2002;3:research0034.1. 12184808 10.1186/gb-2002-3-7-research0034 PMC126239

[13] Danaher P , Warren S , Dennis L , et al. Gene expression markers of tumor infiltrating leukocytes. J Immunother Cancer. 2017;5:18. 28239471 10.1186/s40425-017-0215-8 PMC5319024

[14] Zaidi SH , Harrison TA , Phipps AI , et al. Landscape of somatic mutations in colorectal cancer and the impact on survival. Nat Commun. 2019;11:3644. 10.1038/s41467-020-17386-z PMC7371703 32686686

[15] Therneau T . A Package for Survival Analysis in R. R Package Version 3.2‐13. 2021. Accessed September 1, 2021. https://CRAN.R‐project.org/package=survival

[16] Black A , Gibson TM , Shiels MS , et al. Pooling prospective studies to investigate the etiology of second cancers. Cancer Epidemiol Biomarkers Prev. 2014;23:1598‐1608. 24832874 10.1158/1055-9965.EPI-14-0191 PMC4119533

[17] Benjamini Y , Hochberg Y . Controlling the false discovery rate: a practical and powerful approach to multiple testing. J R Stat Soc Ser B Methodol. 1995;57:289‐300.

[18] Subramanian A , Tamayo P , Mootha VK , et al. Gene set enrichment analysis: a knowledge‐based approach for interpreting genome‐wide expression profiles. Proc Natl Acad Sci USA. 2005;102:15545‐15550. 16199517 10.1073/pnas.0506580102 PMC1239896

[19] Benjamini Y , Yekutieli D . The control of the false discovery rate in multiple testing under dependency. Ann Stat. 2001;29:1165‐1188.

[20] Rozeman EA , Hoefsmit EP , Reijers ILM , et al. Survival and biomarker analyses from the OpACIN‐neo and OpACIN neoadjuvant immunotherapy trials in stage III melanoma. Nat Med. 2021;27:256‐263. 33558721 10.1038/s41591-020-01211-7

[21] Litchfield K , Reading JL , Puttick C , et al. Meta‐analysis of tumor‐ and T cell‐intrinsic mechanisms of sensitization to checkpoint inhibition. Cell. 2021;184:596‐614.e14. 33508232 10.1016/j.cell.2021.01.002 PMC7933824

[22] Grasso CS , Giannakis M , Wells DK , et al. Genetic mechanisms of immune evasion in colorectal cancer. Cancer Discov. 2018;8:730‐749. 29510987 10.1158/2159-8290.CD-17-1327 PMC5984687

[23] Kalbasi A , Ribas A . Tumour‐intrinsic resistance to immune checkpoint blockade. Nat. Rev. Immunol. 2020;20:25‐39. 31570880 10.1038/s41577-019-0218-4 PMC8499690

[24] Spranger S , Bao R , Gajewski TF . Melanoma‐intrinsic β‐catenin signalling prevents anti‐tumour immunity. Nature. 2015;523:231‐235. 25970248 10.1038/nature14404

[25] Todd JJ , Sagar V , Lawal TA , et al. Correlation of phenotype with genotype and protein structure in RYR1‐related disorders. J Neurol. 2018;265:2506‐2524. 30155738 10.1007/s00415-018-9033-2 PMC6182665

[26] Vukcevic M , Zorzato F , Keck S , et al. Gain of function in the immune system caused by a ryanodine receptor 1 mutation. J Cell Sci. 2013;126:3485‐3492. 23704352 10.1242/jcs.130310 PMC3730249

[27] Tate JG , Bamford S , Jubb HC , et al. COSMIC: the catalogue of somatic mutations in cancer. Nucleic Acids Res. 2019;47:D941‐D947. 30371878 10.1093/nar/gky1015 PMC6323903

[28] Maetzel D , Denzel S , Mack B , et al. Nuclear signalling by tumour‐associated antigen EpCAM. Nat Cell Biol. 2009;11:162‐171. 19136966 10.1038/ncb1824

[29] Dai M , Yuan F , Fu C , Shen G , Hu S , Shen G . Relationship between epithelial cell adhesion molecule (EpCAM) overexpression and gastric cancer patients: a systematic review and meta‐analysis. PLoS One. 2017;12:e0175357. 28403178 10.1371/journal.pone.0175357 PMC5389808

[30] Uhlén M , Fagerberg L , Hallström BM , et al. Tissue‐based map of the human proteome. Science. 2015;347:1260419. 25613900 10.1126/science.1260419

[31] Went P , Vasei M , Bubendorf L , et al. Frequent high‐level expression of the immunotherapeutic target Ep‐CAM in colon, stomach, prostate and lung cancers. Br J Cancer. 2006;94:128‐135. 16404366 10.1038/sj.bjc.6602924 PMC2361083

[32] Keller L , Werner S , Pantel K . Biology and clinical relevance of EpCAM. Cell Stress. 2019;3:165‐180. 31225512 10.15698/cst2019.06.188 PMC6558934

[33] Brunet A , Bonni A , Zigmond MJ , et al. Akt promotes cell survival by phosphorylating and inhibiting a Forkhead transcription factor. Cell. 1999;96:857‐868. 10102273 10.1016/s0092-8674(00)80595-4

[34] Chen H‐N , Liang K‐H , Lai J‐K , et al. EpCAM signaling promotes tumor progression and protein stability of PD‐L1 through the EGFR pathway. Cancer Res. 2020;80:5035‐5050. 32978170 10.1158/0008-5472.CAN-20-1264

[35] Liang K‐H , Tso H‐C , Hung S‐H , et al. Extracellular domain of EpCAM enhances tumor progression through EGFR signaling in colon cancer cells. Cancer Lett. 2018;433:165‐175. 29981429 10.1016/j.canlet.2018.06.040

[36] Study of Adecatumumab Relative to FOLFOX After R0 Resection of Colorectal Liver Metastases. ClinicalTrials.gov identifier: NCT00866944. 2011. Accessed March 1, 2022. https://clinicaltrials.gov/ct2/show/NCT00866944

[37] Edrecolomab in Treating Patients With Stage II Colon Cancer. ClinicalTrials.gov identifier: NCT00002968. 2013. Accessed March 2, 2022. https://clinicaltrials.gov/ct2/show/NCT00002968

[38] Schwartzberg LS . Clinical experience with edrecolomab: a monoclonal antibody therapy for colorectal carcinoma. Crit Rev Oncol Hematol. 2001;40:17‐24. 11578913 10.1016/s1040-8428(01)00131-7

[39] Boesch M , Spizzo G , Seeber A . Concise review: aggressive colorectal cancer: role of epithelial cell adhesion molecule in cancer stem cells and epithelial‐to‐mesenchymal transition. Stem Cells Transl Med. 2018;7:495‐501. 29667344 10.1002/sctm.17-0289 PMC5980125

[40] Hamidi H , Ivaska J . Every step of the way: integrins in cancer progression and metastasis. Nat Rev Cancer. 2018;18:533‐548. 30002479 10.1038/s41568-018-0038-z PMC6629548

[41] Kechagia JZ , Ivaska J , Roca‐Cusachs P . Integrins as biomechanical sensors of the microenvironment. Nat Rev Mol Cell Biol. 2019;20:457‐473. 31182865 10.1038/s41580-019-0134-2

[42] Wu A , Zhang S , Liu J , et al. Integrated analysis of prognostic and immune associated integrin family in ovarian cancer. Front Genet. 2020;11:705. 32765584 10.3389/fgene.2020.00705 PMC7379341

[43] Dydensborg AB , Teller IC , Basora N , et al. Differential expression of the integrins alpha6Abeta4 and alpha6Bbeta4 along the crypt‐villus axis in the human small intestine. Histochem Cell Biol. 2009;131:531‐536. 19107504 10.1007/s00418-008-0547-z

[44] Russo A , Saide A , Cagliani R , Cantile M , Botti G , Russo G . rpL3 promotes the apoptosis of p53 mutated lung cancer cells by down‐regulating CBS and NFκB upon 5‐FU treatment. Sci. Rep. 2016;6:38369. 27924828 10.1038/srep38369 PMC5141482

[45] Kang H , Choi MC , Kim S , et al. USP19 and RPL23 as candidate prognostic markers for advanced‐stage high‐grade serous ovarian carcinoma. Cancers (Basel). 2021;13:3976. 34439131 10.3390/cancers13163976 PMC8391231

[46] Latasa MU , Salis F , Urtasun R , et al. Regulation of amphiregulin gene expression by β‐catenin signaling in human hepatocellular carcinoma cells: a novel crosstalk between FGF19 and the EGFR system. PLoS One. 2012;7:e52711. 23285165 10.1371/journal.pone.0052711 PMC3527604

[47] Ortega P , Morán A , de Juan C , et al. Differential Wnt pathway gene expression and E‐cadherin truncation in sporadic colorectal cancers with and without microsatellite instability. Clin Cancer Res. 2008;14:995‐1001. 18281531 10.1158/1078-0432.CCR-07-1588

[48] Cheng X , Xu X , Chen D , Zhao F , Wang W . Therapeutic potential of targeting the Wnt/β‐catenin signaling pathway in colorectal cancer. Biomed. Pharmacother. 2019;110:473‐481. 30530050 10.1016/j.biopha.2018.11.082

[49] Zhang L , Zhao Y , Dai Y , et al. Immune landscape of colorectal cancer tumor microenvironment from different primary tumor location. Front Immunol. 2018;9:1578. 30042763 10.3389/fimmu.2018.01578 PMC6048410

