# Supplementary material for: T cell‐inflamed gene expression profile is associated with favorable disease‐specific survival in non‐hypermutated microsatellite‐stable colorectal cancer patients
Source: Cancer Med. 2022 Nov 7;12(6):6583–93. doi: 10.1002/cam4.5429 (PMC10067089; doi:10.1002/cam4.5429)
Supplement: Supplementary file 1 — Appendix S1 [file CAM4-12-6583-s001.docx]

**Expression of T cell-inflamed gene expression profile is related to favorable disease-specific survival and targeted somatic mutations in colorectal cancer**

**Supplementary Tables and Figures**

| **Table S1. Full list of genes included in 25 pathways used for gene set enrichment analysis** **^†^** | | | | | | | | | |
| --- | --- | --- | --- | --- | --- | --- | --- | --- | --- |
| Pathway names and genes include in the pathway | | | | | | | | | |
| Angiogenesis | | | | | | | | | |
|  | ANGPT1 | ANGPT2 | ANGPTL4 | CCND2 | CCNE1 | CES3 | DLL4 | E2F3 | EDN1 |
|  | EZH2 | FGF18 | FGFR1 | JAG1 | MMP9 | MMRN2 | NFIL3 | PDGFB | PGPEP1 |
|  | RPL7A | SERPINB5 | SERPINH1 | STC1 | THBS1 | TNFAIP6 | FLT1 | FSTL3 | HEY1 |
|  | ITGAV | ITPK1 | TPM1 | TYMP | VCAN | VEGFA | VEGFB | VEGFC |  |
| Antigen Presentation | | | | | | | | | |
|  | ATF3 | B2M | BATF3 | CCL4 | CCR5 | CD1C | CD36 | CD4 | CD74 |
|  | CD8A | CD8B | CDC20 | VHL | CTSS | CTSW | CXCL1 | CYBB | DTX3L |
|  | FCGR1A | HLA.A | HLA.B | HLA.C | HLA.DMA | HLA.DMB | HLA.DOA | HLA.DOB | HLA.DPA1 |
|  | HLA.DPB1 | HLA.DQA1 | HLA.DQA2 | HLA.DQB1 | HLA.DRA | HLA.DRB1 | HLA.DRB5 | HLA.E | HLA.F |
|  | IFNG | IRF8 | ITGAV | KIF2C | KIR2DL3 | KIR3DL1 | KIR3DL2 | KLRD1 | MRC1 |
|  | PSMB10 | PSMB5 | PSMB8 | PSMB9 | PSMC4 | SOCS1 | TAP1 | TAP2 | TAPBP |
|  | THBD | TNF | TRIM21 | UBA7 | UBB | UBE2C | ULBP2 |  |  |
| Apoptosis | | | | | | | | | |
|  | AKT1 | APC | BAD | BAX | BBC3 | BCL2 | BCL2L1 | BCL6B | BID |
|  | BIRC3 | BIRC5 | BLM | CASP1 | CASP3 | CASP8 | CASP9 | CD14 | CDH1 |
|  | CTNNB1 | FADD | GZMB | HMGB1 | LY96 | PSMB10 | PSMB5 | PSMB8 | PSMB9 |
|  | PSMC4 | RIPK1 | RIPK3 | ROCK1 | TICAM1 | TLR3 | TLR4 | TP53 | TNFSF10 |
|  | UBB | TNFRSF10B | |  |  |  |  |  |  |
| Autophagy | | | | | | | | | |
|  | AKT1 | BAD | BCL2 | BCL2L1 | BNIP3 | DEPTOR | HIF1A | HMGB1 | HRAS |
|  | KRAS | MAP3K7 | MAPK10 | MTOR | NRAS | PIK3CA | PIK3CD | PIK3R1 | PIK3R2 |
|  | PRKAA2 | PRKACB | PTEN | RPS6KB1 | RPTOR |  |  |  |  |
| Cell Proliferation | | | | | | | | | |
|  | ANLN | ATM | BIRC5 | BLM | BRCA1 | BRCA2 | CC1 | CCNB1 | CCND1 |
|  | CCND2 | CCND3 | CCNE1 | CCNO | CDC20 | CDC25C | CDK2 | CDK6 | CDKN1A |
|  | CDKN1C | CDKN2A | CDKN2B | CENPF | CEP55 | E2F3 | EXO1 | H2AFX | KIF2C |
|  | MELK | MKI67 | MLH1 | MYC | NBN | PIAS4 | POLD1 | PRKCA | PSMB10 |
|  | PSMB5 | PSMB8 | PSMB9 | PSMC4 | RAD50 | RAD51 | RAD51C | RB1 | RBL2 |
|  | RRM2 | TP53 | TYMS | UBB | UBE2C |  |  |  |  |
| Costimulatory Signaling | | | | | | | | | |
|  | ADORA2A | AKT1 | BTLA | CD2 | CD247 | CD27 | CD274 | CD28 | CD3D |
|  | CD3E | CD3G | CD4 | CD40 | CD40LG | CD44 | CD48 | CD69 | CD70 |
|  | CD80 | CD86 | CHUK | CTLA4 | DPP4 | EGR1 | FYN | HAVCR2 | HLA.DPA1 |
|  | HLA.DPB1 | HLA.DQA1 | HLA.DQA2 | HLA.DQB1 | HLA.DRA | HLA.DRB1 | HLA.DRB5 | ICOS | ICOSLG |
|  | IKBKB | IKBKG | IL15 | IL17A | IL18 | IL18R1 | IL2 | IL2RA | IL2RB |
|  | IL2RG | IL4 | LAG3 | LCK | LILRB2 | LY9 | MAP3K7 | MAP3K8 | MTOR |
|  | NECTIN2 | NFATC2 | NFKB1 | NFKBIA | PDCD1 | PDCD1LG2 | PIK3CA | PIK3R1 | PIK3R2 |
|  | PRR5 | PSMB10 | PSMB5 | PSMB8 | PSMB9 | PSMC4 | PTEN | PTGS2 | PTPN11 |
|  | PTPRC | PVRIG | RELA | RICTOR | RIPK2 | SPP1 | STAT4 | TIGIT | TNFRSF14 |
|  | TNFRSF25 | TNFSF9 | TRAT1 | TSLP | UBB | VTCN1 | ZAP70 |  |  |
| Cytokine and Chemokine Signaling | | | | | | | | | |
|  | AKT1 | CCL13 | CCL14 | CCL18 | CCL19 | CCL2 | CCL20 | CCL21 | CCL22 |
|  | CCL3.L1 | CCL4 | CCL5 | CCL7 | CCL8 | CCR2 | CCR4 | CCR5 | CHUK |
|  | CSF1 | CSF1R | CSF2 | CSF2RB | CSF3 | CSF3R | CX3CL1 | CX3CR1 | CXCL1 |
|  | CXCL10 | CXCL11 | CXCL12 | CXCL13 | CXCL14 | CXCL16 | CXCL2 | CXCL3 | CXCL5 |
|  | CXCL6 | CXCL8 | CXCL9 | CXCR2 | CXCR3 | CXCR4 | CXCR6 | GNG4 | HCK |
|  | HRAS | IKBKB | IKBKG | IL10 | IL10RA | IL11 | IL11RA | IL12RB2 | IL15 |
|  | IL16 | IL17A | IL18 | IL18R1 | IL1A | IL1B | IL1R2 | IL1RN | IL2 |
|  | IL21R | IL22RA1 | IL24 | IL2RA | IL2RB | IL2RG | IL32 | IL33 | IL34 |
|  | IL4 | IL6 | IL6R | IL7R | JAK2 | JAK3 | KRAS | NFKB1 | NFKBIA |
|  | NRAS | PF4 | PIK3CA | PIK3CD | PIK3CG | PIK3R1 | PIK3R2 | PIK3R5 | PRKACB |
|  | RELA | ROCK1 | SHC2 | STAT1 | STAT2 | STAT3 |  |  |  |
| Cytotoxicity | | | | | | | | | |
|  | BBC3 | CBLC | CD47 | CNTFR | FASLG | GHR | GNLY | GZMA | GZMB |
|  | GZMH | GZMK | GZMM | IFI16 | IFI27 | IFI35 | IFI6 | IFIH1 | IFIT1 |
|  | IFIT2 | IFIT3 | IFITM1 | IFITM2 | IFNG | IGF2R | IL11RA | IL12RB2 | IL22RA1 |
|  | IRF1 | IRF4 | IRF9 | ISG15 | JAK1 | JAK2 | JAK3 | KIR2DL3 | KIR3DL1 |
|  | KIR3DL2 | KLRB1 | KLRD1 | KLRK1 | LIF | MX1 | OAS1 | OAS2 | OAS3 |
|  | PRF1 | PRLR | SIRPA | SPRY4 | STAT1 | STAT2 | TNFSF10 |  |  |
| DNA Damage Repair | | | | | | | | | |
|  | ATM | BLM | BRCA1 | BRCA2 | BRIP1 | CC1 | CCNO | CDK2 | DDB2 |
|  | ERCC3 | EXO1 | FANCA | H2AFX | ISG15 | MGMT | MLH1 | MSH2 | MSH6 |
|  | NBN | NEIL1 | PARP4 | PIAS4 | PMS2 | POLD1 | POLR2A | RAD50 | RAD51 |
|  | RAD51C | TNKS | TP53 | UBA7 | UBB | UBE2T | XCL1.2 |  |  |
| Epigenetic Regulation | | | | | | | | | |
|  | ARID1A | BNIP3 | BRD3 | BRD4 | CCND1 | DNMT1 | EZH2 | H2AFX | HDAC11 |
|  | HDAC3 | HDAC4 | HDAC5 | HELLS | HMGA1 | JAK2 | KAT2B | MAP3K12 |  |
| Hedgehog Signaling | | | | | | | | | |
|  | BMP2 | GAS1 | GLI1 | IHH | PRKACB | PRKX | PSMB10 | PSMB5 | PSMB8 |
|  | PSMB9 | PSMC4 | UBB | WNT10A | WNT11 | WNT2 | WNT2B | WNT3A | WNT4 |
|  | WNT5A | WNT5B | WNT7B |  |  |  |  |  |  |
| Hypoxia | | | | | | | | | |
|  | AKT1 | ALDOA | ANGPT1 | ANGPT2 | BCL2 | CDKN1A | CYBB | EDN1 | EGF |
|  | EGFR | EIF4EBP1 | ENO1 | ERBB2 | FLT1 | HIF1A | HK1 | HK2 | IFNG |
|  | IFNGR1 | IFNGR2 | IL6 | IL6R | LDHA | MTOR | NFKB1 | NOS2 | PDK1 |
|  | PFKFB3 | PIK3CA | PIK3CD | PIK3CG | PIK3R1 | PIK3R2 | PIK3R5 | PRKCA | RELA |
|  | RPS6KB1 | SLC2A1 | STAT3 | TFRC | TLR4 | VEGFA | VHL |  |  |
| Immune Cell Adhesion and Migration | | | | | | | | | |
|  | CD2 | CD274 | CD276 | CD28 | CD4 | CD40 | CD40LG | CD58 | CD6 |
|  | CD80 | CD86 | CD8A | CD8B | CDH1 | CDH2 | CDH5 | CLEC14A | CLEC4E |
|  | CLEC5A | CLEC7A | CLECL1 | CTLA4 | CTNNB1 | CXCL12 | CXCR4 | CYBB | HLA.A |
|  | HLA.B | HLA.C | HLA.DMA | HLA.DMB | HLA.DOA | HLA.DOB | HLA.DPA1 | HLA.DPB1 | HLA.DQA1 |
|  | HLA.DQA2 | HLA.DQB1 | HLA.DRA | HLA.DRB1 | HLA.DRB5 | HLA.E | HLA.F | ICAM1 | ICAM2 |
|  | ICAM3 | ICAM5 | ICOS | ICOSLG | ITGA1 | ITGA2 | ITGA4 | ITGA6 | ITGAE |
|  | ITGAL | ITGAM | ITGAV | ITGAX | ITGB2 | ITGB3 | ITGB8 | MMP9 | NCAM1 |
|  | NECTIN1 | NECTIN2 | PDCD1 | PDCD1LG2 | PECAM1 | PIK3CA | PIK3CD | PIK3CG | PIK3R1 |
|  | PIK3R2 | PIK3R5 | PRKCA | PTPN11 | PTPRC | PVR | ROCK1 | SELE | SELL |
|  | SELP | SIGLEC1 | THY1 | TIGIT | VCAM1 | VCAN | VTCN1 |  |  |
| Interferon Signaling | | | | | | | | | |
|  | B2M | CD44 | EGR1 | EIF2AK2 | FCGR1A | FLNB | GBP1 | GBP2 | GBP4 |
|  | GHR | HLA.A | HLA.B | HLA.C | HLA.DPA1 | HLA.DPB1 | HLA.DQA1 | HLA.DQA2 | HLA.DQB1 |
|  | HLA.DRA | HLA.DRB1 | HLA.DRB5 | HLA.E | HLA.F | ICAM1 | IFI16 | IFI27 | IFI35 |
|  | IFI6 | IFIH1 | IFIT1 | IFIT2 | IFIT3 | IFITM1 | IFITM2 | IF1 | IFR1 |
|  | IFNG | IFNGR1 | IFNGR2 | IGF2R | IRF1 | IRF2 | IRF3 | IRF4 | IRF5 |
|  | IRF7 | IRF8 | IRF9 | ISG15 | JAK1 | JAK2 | MX1 | NCAM1 | OAS1 |
|  | OAS2 | OAS3 | OASL | PSMB8 | PTPN11 | RSAD2 | SOCS1 | STAT1 | STAT2 |
|  | TRIM21 | UBA7 | UBB | VCAM1 |  |  |  |  |  |
| JAK-STAT Signaling | | | | | | | | | |
|  | AKT1 | BCL2 | BCL2L1 | CCND1 | CCND2 | CCND3 | CDKN1A | CNTFR | CSF2 |
|  | CSF2RB | CSF3 | CSF3R | GHR | HRAS | IF1 | IFR1 | IFNG | IFNGR1 |
|  | IFNGR2 | IL10 | IL10RA | IL11 | IL11RA | IL12RB2 | IL15 | IL2 | IL21R |
|  | IL22RA1 | IL24 | IL2RA | IL2RB | IL2RG | IL4 | IL6 | IL6R | IL7R |
|  | IRF9 | JAK1 | JAK2 | JAK3 | LIF | MTOR | MYC | PIAS4 | PIK3CA |
|  | PIK3CD | PIK3CG | PIK3R1 | PIK3R2 | PIK3R5 | PRLR | PTPN11 | SOCS1 | STAT1 |
|  | STAT2 | STAT3 | STAT4 | TSLP |  |  |  |  |  |
| Lymphoid Compartment | | | | | | | | | |
|  | BLK | CCR4 | CD19 | CD1C | CD2 | CD27 | CD274 | CD28 | CD38 |
|  | CD3D | CD3E | CD3G | CD40 | CD40LG | CD48 | CD5 | CD6 | CD7 |
|  | CD70 | CD79A | CD79B | CD80 | CD86 | CD8A | CD8B | CD96 | CTLA4 |
|  | CX3CL1 | CXCL10 | CXCL11 | CXCL13 | CXCL16 | CXCL9 | CXCR3 | DPP4 | EGR1 |
|  | EOMES | F2RL1 | GNLY | GZMA | GZMB | GZMH | GZMK | GZMM | HLA.DOB |
|  | ICOS | ICOSLG | IDO1 | IFI27 | IFIT1 | IFITM1 | IFNG | IGF2R | IL11 |
|  | IL12RB2 | IL18R1 | IL2RG | IRF4 | IRF9 | ISG15 | ITGA1 | JAK1 | JAK2 |
|  | KIR2DL3 | KIR3DL1 | KIR3DL2 | KLRB1 | KLRD1 | KLRK1 | LAG3 | LCK | LY9 |
|  | MS4A1 | MX1 | PDCD1 | PRF1 | PVR | SLAMF7 | STAT1 | STAT2 | TBX21 |
|  | TIGIT | TNFRSF25 | ZAP70 |  |  |  |  |  |  |
| MAPK | | | | | | | | | |
|  | AKT1 | ANGPT1 | ANGPT2 | BAD | BCL2L1 | CASP3 | CD14 | CHUK | CSF1 |
|  | CSF1R | DUSP1 | DUSP2 | DUSP5 | EGF | EGFR | FAS | FASLG | FGF13 |
|  | FGF18 | FGF9 | FGFR1 | FLNB | FLT1 | GNG4 | HRAS | IKBKB | IKBKG |
|  | IL1A | IL1B | IL1R2 | KDR | KIT | KRAS | MAP3K12 | MAP3K5 | MAP3K7 |
|  | MAP3K8 | MAPK10 | MET | MYC | NF1 | NFKB1 | NFKB2 | NGFR | NRAS |
|  | PDGFA | PDGFB | PDGFRB | PIK3CA | PIK3CD | PIK3CG | PIK3R1 | PIK3R2 | PIK3R5 |
|  | PLA1A | PLA2G2A | PRKACB | PRKCA | PTPN11 | RASAL1 | RASGRF1 | RELA | RELB |
|  | SHC2 | TGFB1 | TGFB2 | TGFB3 | TGFBR1 | TGFBR2 | TNF | TNFRSF1A | TP53 |
|  | VEGFA | VEGFB | VEGFC | ZAP70 |  |  |  |  |  |
| Matrix Remodeling and Metastasis | | | | | | | | | |
|  | A2M | BMP2 | CASP3 | CD36 | CD44 | CD47 | CDH1 | COL11A1 | COL11A2 |
|  | COL17A1 | COL4A5 | COL5A1 | COL6A3 | COMP | CTSS | ICAM1 | ICAM2 | ICAM3 |
|  | ITGA1 | ITGA2 | ITGA4 | ITGA6 | ITGAE | ITGAL | ITGAM | ITGAV | ITGAX |
|  | ITGB2 | ITGB3 | ITGB8 | KDR | LAMA1 | LAMB3 | LAMC2 | LOXL2 | LTBP1 |
|  | MMP1 | MMP7 | MMP9 | NCAM1 | NID2 | PDGFA | PDGFB | PECAM1 | PLOD2 |
|  | PRKCA | RELN | SERPINH1 | SPP1 | TGFB1 | TGFB2 | TGFB3 | THBS1 | VCAM1 |
|  | VCAN |  |  |  |  |  |  |  |  |
| Metabolic Stress | | | | | | | | | |
|  | AKT1 | AQP9 | ATM | CC1 | CCNE1 | CD300A | CDK2 | CDK6 | CDKN1A |
|  | CDKN2A | CDKN2B | CEBPB | CXCL8 | DEPTOR | E2F3 | EGFR | EIF4EBP1 | ENO1 |
|  | ERBB2 | ERO1A | EZH2 | FBP1 | FGFR1 | G6PD | GLS | GOT1 | GOT2 |
|  | H2AFX | HIF1A | HK1 | HK2 | HMGA1 | HRAS | IKBKB | IL1A | IL6 |
|  | KIT | KRAS | LDHA | LDHB | MAP3K5 | MAPK10 | MET | MTOR | MYC |
|  | NBN | NFKB1 | NRAS | PC | PCK2 | PDGFRB | PDK1 | PFKFB3 | PFKM |
|  | PIK3CA | PIK3CD | PIK3CG | PIK3R1 | PIK3R2 | PIK3R5 | PKM | PRKAA2 | PRKCA |
|  | PRR5 | PTEN | RAD50 | RB1 | RELA | RICTOR | RPS6KB1 | RPTOR | SGK1 |
|  | SLC16A1 | SLC1A5 | SLC2A1 | SLC7A5 | STAT3 | TNF | TP53 | TPI1 | UBB |
|  | UBE2C | VEGFA | VHL |  |  |  |  |  |  |
| Myeloid Compartment | | | | | | | | | |
|  | ANGPT1 | AREG | ARG1 | C5AR1 | CCL2 | CCL20 | CCL4 | CCL5 | CCL8 |
|  | CD14 | CD47 | CDKN1A | CEBPB | CLEC4E | CLEC5A | CLEC7A | COL11A1 | COL17A1 |
|  | CRABP2 | CSF1 | CSF1R | CSF3R | CXCL1 | CXCL12 | CXCL2 | CXCL3 | CXCL5 |
|  | CXCL6 | CYBB | DAB2 | DLL4 | FCAR | FCGR1A | FCN1 | FOSL1 | FPR1 |
|  | FPR3 | HCK | IER3 | IL1A | IL1B | IL1RN | ITGAM | ITGAX | LAMB3 |
|  | LIF | LILRA5 | LILRB2 | LY96 | LYZ | MARCO | MMP1 | MRC1 | NFAM1 |
|  | NLRP3 | NOS2 | OLR1 | P2RY13 | PDZK1IP1 | PTGS2 | S100A12 | S100A8 | S100A9 |
|  | SERPI1 | SIGLEC8 | SIRPA | SIRPB2 | SLC11A1 | TLR1 | TLR2 | TLR4 | TLR8 |
|  | TNFAIP6 | TREM1 | TREM2 |  |  |  |  |  |  |
| NF-kappaB Signaling | | | | | | | | | |
|  | BIRC3 | CD27 | CD40 | CD40LG | CD70 | CHUK | FASLG | IKBKB | IKBKG |
|  | LTB | NFKB1 | NFKB2 | NFKBIA | NFKBIE | PSMB10 | PSMB5 | PSMB8 | PSMB9 |
|  | PSMC4 | RELA | RELB | RELN | TNF | TNFRSF11A | TNFRSF11B | TNFRSF14 | TNFRSF17 |
|  | TNFRSF18 | TNFRSF1A | TNFRSF1B | TNFRSF25 | TNFRSF4 | TNFSF12 | TNFSF13 | TNFSF13B | TNFSF18 |
|  | TNFSF4 | UBB |  |  |  |  |  |  |  |
| Notch Signaling | | | | | | | | | |
|  | APH1B | CCND1 | DLL1 | DLL4 | DTX3L | DTX4 | E2F3 | GZMB | HDAC11 |
|  | HDAC3 | HDAC4 | HDAC5 | HES1 | HEY1 | HIF1A | JAG1 | JAG2 | KAT2B |
|  | MAML2 | MFNG | MYC | NOTCH1 | NOTCH2 | TP53 | UBB |  |  |
| PI3K-Akt | | | | | | | | | |
|  | AKT1 | ANGPT1 | ANGPT2 | BAD | BCL2 | BCL2L1 | BRCA1 | CASP9 | CCND1 |
|  | CCND2 | CCND3 | CCNE1 | CD19 | CDK2 | CDK6 | CDKN1A | CHUK | COL4A5 |
|  | COL6A3 | COMP | CSF1 | CSF1R | CSF3 | CSF3R | EGF | EGFR | EIF4EBP1 |
|  | FASLG | FGF13 | FGF18 | FGF9 | FGFR1 | FLT1 | GHR | GNG4 | HRAS |
|  | IF1 | IKBKB | IKBKG | IL2 | IL2RA | IL2RB | IL2RG | IL4 | IL6 |
|  | IL6R | IL7R | ITGA1 | ITGA2 | ITGA4 | ITGA6 | ITGAV | ITGB3 | ITGB8 |
|  | JAK1 | JAK2 | JAK3 | KDR | KIT | KRAS | LAMA1 | LAMB3 | LAMC2 |
|  | MET | MTOR | MYC | NFKB1 | NGFR | NRAS | PCK2 | PDGFA | PDGFB |
|  | PDGFRB | PIK3CA | PIK3CD | PIK3CG | PIK3R1 | PIK3R2 | PIK3R5 | PRKAA2 | PRKCA |
|  | PRLR | PTEN | RBL2 | RELA | RELN | RPS6KB1 | RPTOR | SGK1 | SPP1 |
|  | SYK | TCL1A | THBS1 | TLR2 | TLR4 | TP53 | VEGFA | VEGFB | VEGFC |
| TGF-beta Signaling | | | | | | | | | |
|  | ACVR1C | BAMBI | BMP2 | CDKN2B | ID4 | IFNG | INHBA | LTBP1 | MYC |
|  | RBL2 | ROCK1 | RPS6KB1 | SMAD5 | TGFB1 | TGFB2 | TGFB3 | TGFBR1 | TGFBR2 |
|  | THBS1 | TNF | UBB |  |  |  |  |  |  |
| Wnt Signaling | | | | | | | | | |
|  | APC | AXIN1 | BAMBI | CCND1 | CCND2 | CCND3 | CTNNB1 | DKK1 | FOSL1 |
|  | FZD8 | FZD9 | GPC4 | MAP3K7 | MAPK10 | MMP7 | MYC | NFATC2 | PRKACB |
|  | PRKCA | SFRP1 | SFRP4 | SOX11 | SOX2 | TP53 | WNT10A | WNT11 | WNT2 |
|  | WNT2B | WNT3A | WNT4 | WNT5A | WNT5B | WNT7B |  |  |  |
| ^†^ Table is modified from functional annotation gene list from Nanostring nCounter IO 360 Panel | | | | | | | | | |

| **Table S2. Associations between continuous standardized T cell-inflamed GEP score and CRC-specific survival after adjustment for cancer stage** | | | | |
| --- | --- | --- | --- | --- |
| Analysis groups | No. cases | No. events | HR (95% CIs) | P-value |
| All cases | 79 | 24 |  |  |
| model 1 |  |  | 0.39 (0.23 - 0.65) | 3.25E-04 |
| model 2 |  |  | 0.47 (0.27 - 0.80) | 0.006 |
|  |  |  |  |  |
| Non-Hypermutated and MSS cases | 60 | 22 |  |  |
| model 1 |  |  | 0.50 (0.29 - 0.87) | 0.015 |
| model 3 |  |  | 0.40 (0.21 - 0.76) | 0.005 |
| Abbreviation: No: number; HR: hazard ratio; CIs: confidence intervals  Grouped stage is a binary variable including a combination of stage I and II (35 cases) and a combination of stage III and IV (44 cases) | | | | |
| model 1: adjusted for age at diagnosis, sex, and grouped stage (I/II vs. III/IV)  model 2: adjusted for age at diagnosis, sex, hypermutation or MSI status, and group stage (I/II vs. III/IV)  model 3: adjusted for age at diagnosis, sex, log-transformed total number of mutations, and grouped stage (I/II vs. III/IV) | | | | |

| **Table S3. Associations between continuous standardized T cell-inflamed GEP score and overall survival** | | | | |
| --- | --- | --- | --- | --- |
| Analysis groups | No. cases | No. events | HR (95% CIs) | P-value |
| All cases | 79 | 46 |  |  |
| model 1 |  |  | 0.49 (0.33 - 0.72) | 3.00E-04 |
| model 2 |  |  | 0.56 (0.37 - 0.85) | 0.00692 |
| model 3 |  |  | 0.49 (0.32 - 0.74) | 8.23E-04 |
| Abbreviation: No: number; HR: hazard ratio; CIs: confidence intervals | | | |  |
| model 1: adjusted for age at diagnosis and sex  model 2: adjusted for age at diagnosis, sex, hypermutation or MSI status  model 3: adjusted for age at diagnosis, sex, log-transformed total number of mutations | | | | |
|  |  |  |  |  |

| **Table S4. Associations between dichotomized T cell-inflamed GEP score and CRC-specific survival** | | | | |
| --- | --- | --- | --- | --- |
| Analysis groups | No. cases | No. events | HR (95% CIs) | P-value |
| All cases | 79 | 24 |  |  |
| model 1 |  |  | 0.12 (0.03 - 0.49) | 3.70E-03 |
| model 2 |  |  | 0.19 (0.04 - 0.81) | 0.0254 |
|  |  |  |  |  |
| Non-Hypermutated and MSS cases | 61 | 23 |  |  |
| model 1 |  |  | 0.23 (0.05 - 0.96) | 0.0443 |
| model 3 |  |  | 0.16 (0.04 - 0.73) | 0.0174 |
| Abbreviation: No: number; HR: hazard ratio; CIs: confidence intervals | | | | |
| model 1: adjusted for age at diagnosis and sex | | | | |
| model 2: adjusted for age at diagnosis, sex, hypermutation and/or MSI status  model 3: adjusted for age at diagnosis, sex, log-transformed total number of mutations | | | |  |

| **Table S5. Association between continuous standardized T cell-inflamed GEP score with somatic mutations (full list)** | | | | | |
| --- | --- | --- | --- | --- | --- |
| Gene/ Pathway | No.carriers | Covariates for adjustment 1 | | Covariates for adjustment 2 | |
|  |  | nominal P-value | adj P-value | nominal P-value | adj P-value |
| *APC* | 44 | 0.010 | 0.054 | 0.093 | 0.465 |
| *TP53* | 41 | 0.764 | 0.950 | 0.286 | 0.544 |
| *KRAS* | 20 | 0.706 | 0.950 | 0.914 | 0.914 |
| *SYNE1* | 16 | 0.040 | 0.119 | 0.199 | 0.544 |
| *RYR1* | 16 | 0.003 | 0.028 | 0.112 | 0.465 |
| *FBXW7* | 11 | 0.942 | 0.953 | 0.297 | 0.544 |
| *SMAD4* | 11 | 0.778 | 0.950 | 0.889 | 0.914 |
| *PIK3CA* | 11 | 0.953 | 0.953 | 0.127 | 0.465 |
| *AMER1* | 10 | 0.045 | 0.119 | 0.442 | 0.635 |
| *ARID1A* | 10 | 0.054 | 0.119 | 0.462 | 0.635 |
| *KMT2C* | 10 | 0.142 | 0.260 | 0.697 | 0.852 |
| WNT | 61 | 0.696 | 0.759 | 0.969 | 0.969 |
| p53 | 44 | 0.759 | 0.759 | 0.327 | 0.544 |
| RTK_RAS | 35 | 0.015 | 0.076 | 0.089 | 0.445 |
| TGF_beta | 26 | 0.095 | 0.239 | 0.625 | 0.782 |
| IGF2_PI3K | 18 | 0.501 | 0.759 | 0.185 | 0.462 |
| Abbreviation: No.: number; adj: adjusted | | |  |  |  |
| Covariates 1 includes age at diagnosis and sex | | |  |  |  |
| Covariates 2 includes age at diagnosis, sex, hypermutation or MSI status | | | | |  |
| Mutated pathway is defined as any mutated (yes/no) genes included in the pathway | | | | | |
| Wnt pathway: *AMER1, APC, ARID1A, AXIN1, AXIN2, CTNNB1, FBXW7, RNF43, SOX9, TCF7, TCF7L2, ZNRF3* | | | | | |
| p53: *ATM, TP53* | |  |  |  |  |
| RTK_RAS: *BRAF, ERBB2, ERBB3, KRAS, NRAS* | | | |  |  |
| TGF_beta: *ACVR1B, ACVR2A, BMPR1A, BMPR2, GDF5, SMAD2, SMAD3, SMAD4, TGFBR1, TGFBR2* | | | | | |
| IGF2_PI3K: *IGF2, PIK3CA, PIK3R1, PTEN* | | |  |  |  |

| **Table S6. Gene set differently enriched for the standardized T-cell inflamed GEP score (full list)** | | | | | | | |  | | | | |  |  |  |
| --- | --- | --- | --- | --- | --- | --- | --- | --- | --- | --- | --- | --- | --- | --- | --- |
|  | Gene set | Gene set size | ES | | NES | | Nominal P-value | | | FDR adjusted P-value | | | |  |  |
| Model 1 | | | |  | |  | | | | |  |  |  |  |  |
| Up-regulation | Lymphoid compartment | 76 | 0.74 | | 1.84 | | <0.001 | | | <0.001^***^ | | | |  |  |
|  | Cytotoxicity | 47 | 0.62 | | 1.5 | | <0.001 | | | 0.012^*^ | | | |  |  |
|  | Costimulatory signaling | 72 | 0.54 | | 1.33 | | 0.007 | | | 0.136 | | | |  |  |
|  | Interferon signaling | 50 | 0.54 | | 1.3 | | 0.025 | | | 0.146 | | | |  |  |
|  | Antigen presentation | 38 | 0.53 | | 1.28 | | 0.055 | | | 0.157 | | | |  |  |
|  | Cytokine and chemokine signaling | 90 | 0.49 | | 1.22 | | 0.04 | | | 0.261 | | | |  |  |
|  | Immune cell adhesion and migration | 70 | 0.49 | | 1.21 | | 0.064 | | | 0.242 | | | |  |  |
|  | JAK-STAT signaling | 49 | 0.48 | | 1.17 | | 0.142 | | | 0.304 | | | |  |  |
|  | NF-kappaB signaling | 34 | 0.47 | | 1.12 | | 0.274 | | | 0.44 | | | |  |  |
|  | Myeloid compartment | 71 | 0.42 | | 1.04 | | 0.407 | | | 0.682 | | | |  |  |
|  | Apoptosis | 36 | 0.31 | | 0.73 | | 0.923 | | | >0.999 | | | |  |  |
|  | Matrix remodeling and metastasis | 54 | 0.18 | | 0.43 | | >0.999 | | | >0.999 | | | |  |  |
|  | Epigenetic regulation | 17 | 0.19 | | 0.41 | | 0.998 | | | >0.999 | | | |  |  |
|  | Hypoxia | 40 | 0.16 | | 0.39 | | >0.999 | | | >0.999 | | | |  |  |
|  | PI3K-AKT | 89 | 0.14 | | 0.35 | | >0.999 | | | >0.999 | | | |  |  |
|  | MAPK | 72 | 0.14 | | 0.35 | | >0.999 | | | >0.999 | | | |  |  |
| Down-regulation | Wnt signaling | 29 | -0.43 | | -1.9 | | <0.001 | | | 0.015^*^ | | | |  |  |
|  | Hedgehog signaling | 18 | -0.4 | | -1.53 | | 0.021 | | | 0.083 | | | |  |  |
|  | TGF-beta signaling | 19 | -0.33 | | -1.29 | | 0.091 | | | 0.247 | | | |  |  |
|  | Metabolic stress | 80 | -0.23 | | -1.27 | | <0.001 | | | 0.211 | | | |  |  |
|  | Notch signaling | 24 | -0.29 | | -1.22 | | 0.137 | | | 0.243 | | | |  |  |
|  | Autophagy | 22 | -0.28 | | -1.15 | | 0.206 | | | 0.314 | | | |  |  |
|  | Cell proliferation | 47 | -0.19 | | -0.98 | | 0.569 | | | 0.641 | | | |  |  |
|  | DNA damage repair | 29 | -0.19 | | -0.86 | | 0.768 | | | 0.838 | | | |  |  |
|  | Angiogenesis | 35 | -0.18 | | -0.85 | | 0.766 | | | 0.761 | | | |  |  |
| Model 2 | | | | | | | |  | | | | |  |  |  |
| Up-regulation | Lymphoid compartment | 72 | 0.76 | 1.77 | | <0.001 | | | <0.001^***^ | | | | | |  |
|  | Cytotoxicity | 45 | 0.58 | 1.33 | | 0.008 | | | 0.113 | | | | | |  |
|  | Interferon signaling | 49 | 0.55 | 1.27 | | 0.022 | | | 0.19 | | | | | |  |
|  | Cytokine and chemokine signaling | 90 | 0.51 | 1.21 | | 0.025 | | | 0.312 | | | | | |  |
|  | Antigen presentation | 36 | 0.52 | 1.17 | | 0.147 | | | 0.421 | | | | | |  |
|  | Immune cell adhesion and migration | 67 | 0.49 | 1.14 | | 0.122 | | | 0.45 | | | | | |  |
|  | Costimulatory signaling | 65 | 0.48 | 1.12 | | 0.18 | | | 0.509 | | | | | |  |
|  | JAK-STAT signaling | 48 | 0.47 | 1.08 | | 0.288 | | | 0.59 | | | | | |  |
|  | NF-kappaB signaling | 33 | 0.47 | 1.05 | | 0.391 | | | 0.664 | | | | | |  |
|  | Myeloid compartment | 71 | 0.4 | 0.94 | | 0.688 | | | >0.999 | | | | | |  |
|  | Apoptosis | 35 | 0.28 | 0.64 | | 0.978 | | | >0.999 | | | | | |  |
|  | Epigenetic regulation | 17 | 0.3 | 0.64 | | 0.95 | | | >0.999 | | | | | |  |
|  | PI3K-AKT | 89 | 0.23 | 0.53 | | >0.999 | | | >0.999 | | | | | |  |
|  | Hypoxia | 40 | 0.23 | 0.53 | | 0.996 | | | >0.999 | | | | | |  |
|  | MAPK | 72 | 0.22 | 0.53 | | >0.999 | | | >0.999 | | | | | |  |
|  | Matrix remodeling and metastasis | 54 | 0.22 | 0.51 | | 0.999 | | | 0.996 | | | | | |  |
| Down-regulation | Cell proliferation | 46 | -0.3 | -1.6 | | <0.001 | | | 0.089 | | | | | |  |
|  | Notch signaling | 24 | -0.27 | -1.15 | | 0.193 | | | 0.899 | | | | | |  |
|  | Metabolic stress | 80 | -0.16 | -1.12 | | <0.001 | | | 0.747 | | | | | |  |
|  | Hedgehog signaling | 17 | -0.24 | -0.91 | | 0.643 | | | >0.999 | | | | | |  |
|  | DNA damage repair | 29 | -0.19 | -0.88 | | 0.705 | | | >0.999 | | | | | |  |
|  | TGF-beta signaling | 19 | -0.2 | -0.81 | | 0.812 | | | >0.999 | | | | | |  |
|  | Wnt signaling | 29 | -0.17 | -0.79 | | 0.893 | | | >0.999 | | | | | |  |
|  | Angiogenesis | 35 | -0.14 | -0.72 | | 0.987 | | | >0.999 | | | | | |  |
|  | Autophagy | 22 | -0.16 | -0.7 | | 0.954 | | | 0.955 | | | | | |  |
| Abbreviation: ES: enrichment score; NES: normalized enrichment score  ^*^ Significant FDR adjusted p-value at the level of 0.05 | | | | | | | | | | | | |  |  |  |
| Model 1: age at diagnosis and sex were adjusted in differential expression analysis | | | | | | | | | | | | | | | |
| Model 2: age at diagnosis, sex, and hypermutation or MSI status were adjusted in differential expression analysis | | | | | | | | | | | | | | | |


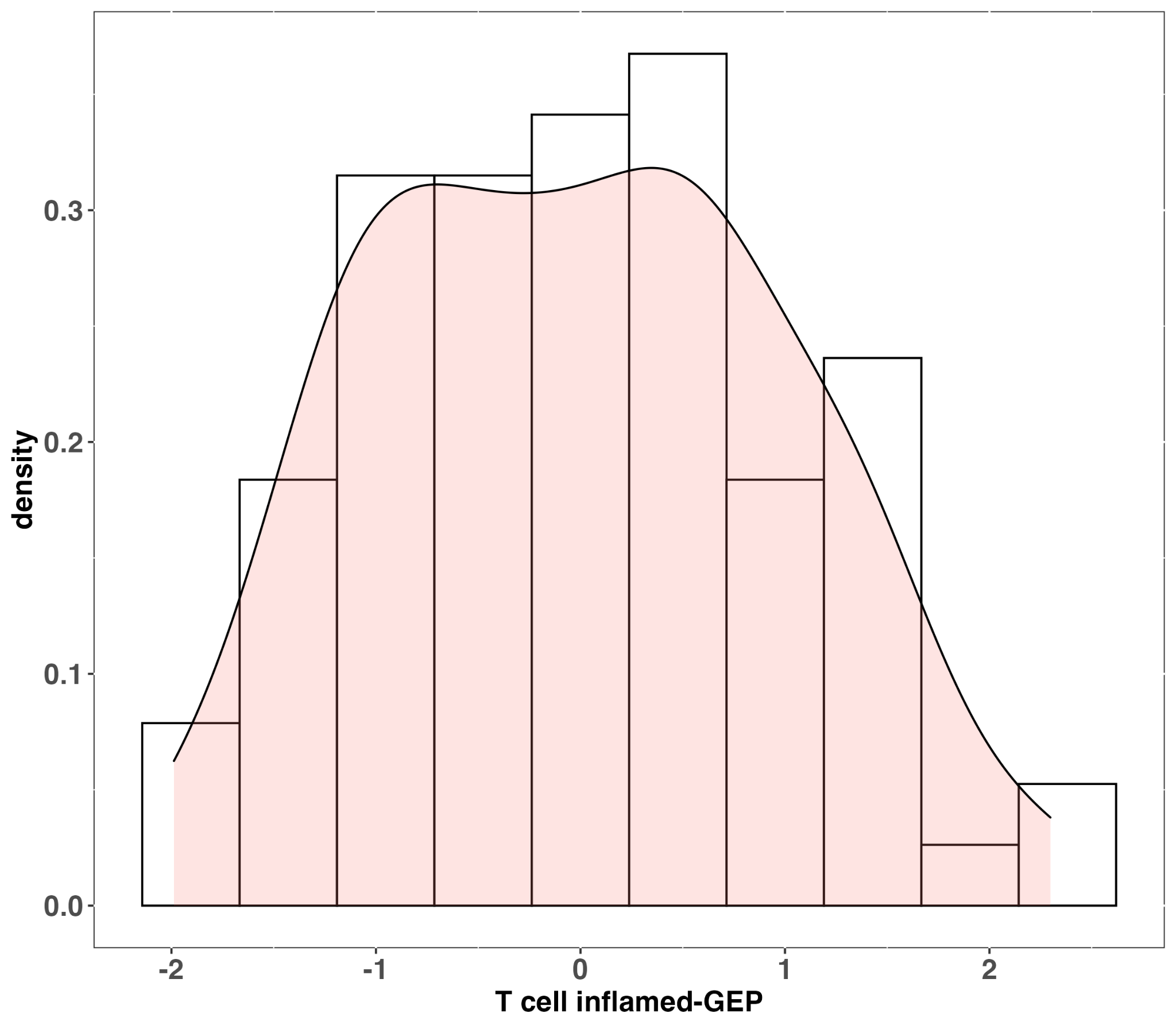


Figure S1. Histogram of T cell inflamed-GEP, with density curve


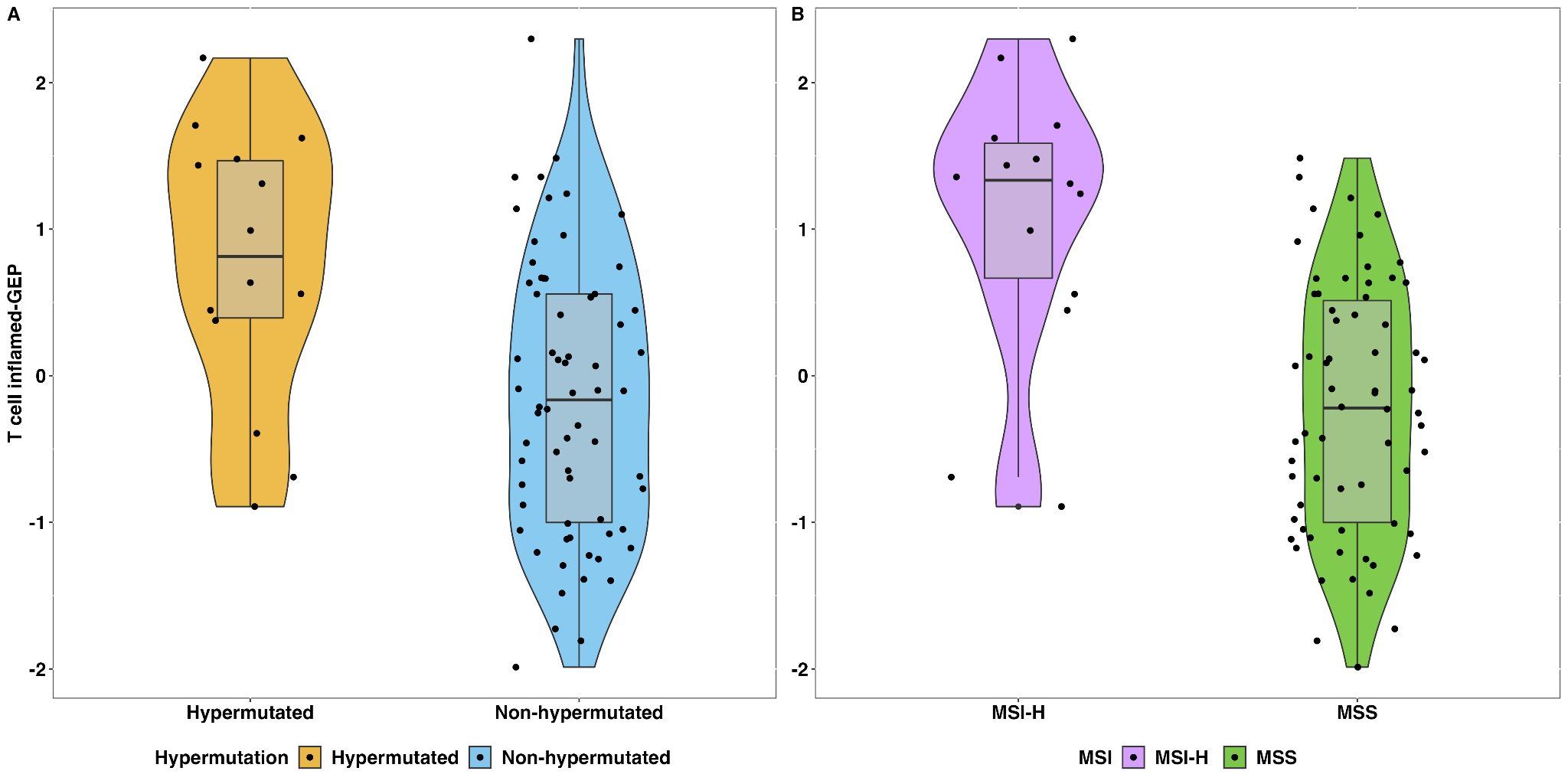


FigureS2. Distribution of standardized T cell inflamed-GEP across (A) hypermutation status and (B) MSI status


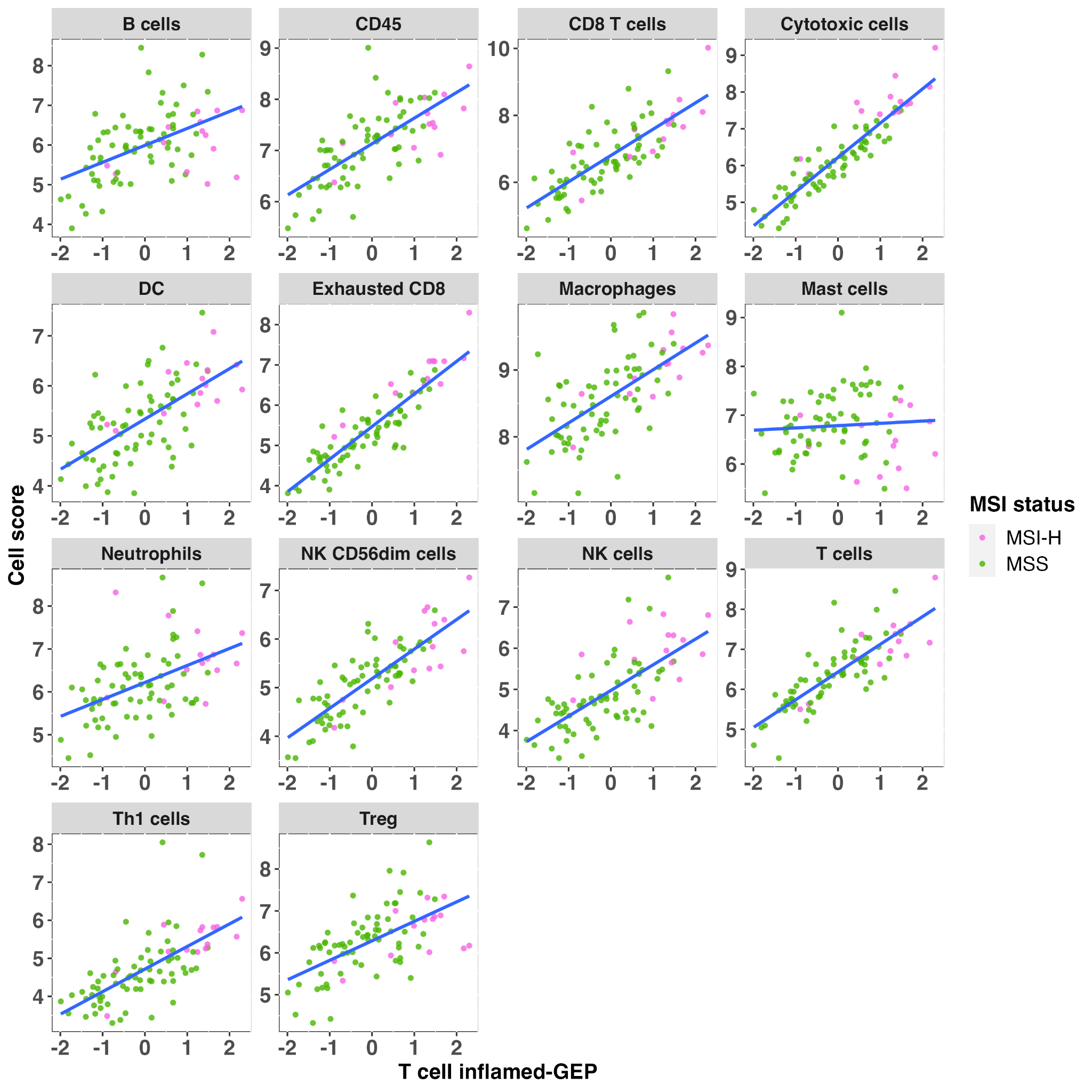


Figure S3. Correlation between immune cell scores and T cell inflamed-GEP, per cell type

| **A** | 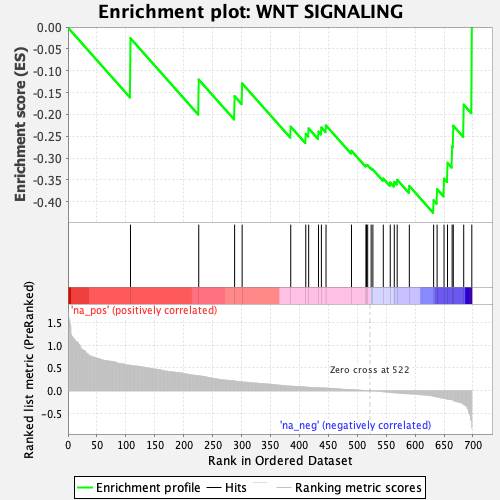 | **B** | 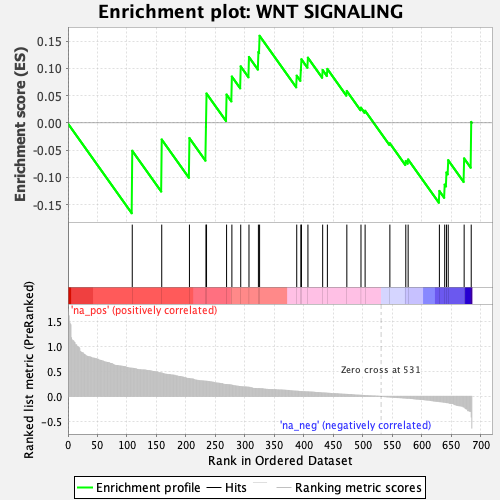 |
| --- | --- | --- | --- |

Figure S4. Enrichment plot for Wnt signaling: (A) age at diagnosis and sex were adjusted in differential expression analysis; (B) Model 2: age at diagnosis, sex, and hypermutation or MSI status were adjusted in differential expression analysis. It showed “na_pos” and “na_neg” because we used continuous T cell-inflamed GEP.
